# Tungsten Enzyme Using Hydrogen as an Electron Donor to Reduce Carboxylic Acids and NAD^+^

**DOI:** 10.1021/acscatal.2c02147

**Published:** 2022-07-06

**Authors:** Agnieszka Winiarska, Dominik Hege, Yvonne Gemmecker, Joanna Kryściak-Czerwenka, Andreas Seubert, Johann Heider, Maciej Szaleniec

**Affiliations:** † Jerzy Haber Institute of Catalysis and Surface Chemistry Polish Academy of Sciences, Kraków 30-239, Poland; ‡ Faculty of Biology, Philipps-Universität Marburg, Marburg D-35043, Germany; § Faculty of Chemistry, Philipps-Universität Marburg, Marburg D-35043, Germany; ∥ Center for Synthetic Microbiology, Philipps-Universität Marburg, Marburg D-35043, Germany

**Keywords:** oxidoreductases, metalloenzymes, tungsten, aldehydes, biotransformations

## Abstract

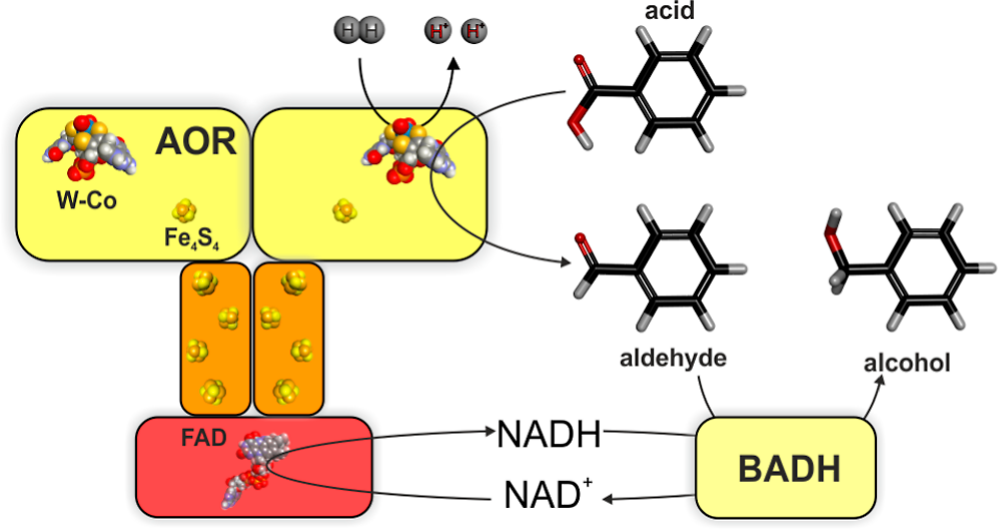

Tungsten-dependent aldehyde oxidoreductases (AORs) catalyze the oxidation of aldehydes to acids and are the only known enzymes reducing non-activated acids using electron donors with low redox potentials. We report here that AOR from *Aromatoleum aromaticum* (AOR_*Aa*_) catalyzes the reduction of organic acids not only with low-potential Eu(II) or Ti(III) complexes but also with H_2_ as an electron donor. Additionally, AOR_*Aa*_ catalyzes the H_2_-dependent reduction of NAD^+^ or benzyl viologen. The rate of H_2_-dependent NAD^+^ reduction equals to 10% of that of aldehyde oxidation, representing the highest H_2_ turnover rate observed among the Mo/W enzymes. As AOR_*Aa*_ simultaneously catalyzes the reduction of acids and NAD^+^, we designed a cascade reaction utilizing a NAD(P)H-dependent alcohol dehydrogenase to reduce organic acids to the corresponding alcohols with H_2_ as the only reductant. The newly discovered W-hydrogenase side activity of AOR_*Aa*_ may find applications in either NADH recycling or conversion of carboxylic acids to more useful biochemicals.

## Significance

Hydrogen is simultaneously a clean fuel for energy generation and a cheap reductant for catalytic transformation processes producing useful compounds from biomass. We characterize here a novel hydrogenase functionality in a tungsten-containing enzyme, aldehyde oxidoreductase (AOR) from *Aromatoleum aromaticum* (*A. aromaticum*) (AOR_*Aa*_). In addition to the standard reactivity of reversible aldehyde oxidation, AOR_*Aa*_ also accepts hydrogen as an electron donor for the reduction of either organic acids or NAD^+^, which can be used to produce valuable aldehydes or to recycle NADH in biochemical cascade reactions. We provide examples for coupling the enzyme to industrially important NADH-dependent dehydrogenases that are already used in the fine chemical and pharmaceutical industry, as well as for converting carboxylic acids to alcohols by coupling AOR_*Aa*_ with appropriate alcohol dehydrogenases.

## Introduction

Tungsten (W) exhibits similar chemical properties to molybdenum (Mo), and both occur in most environments in the form of the oxyanions tungstate (WO_4_^2–^) and molybdate (MoO_4_^2–^).^1,2^ Both elements are used in biology as constituents of the so-called Mo- or W-cofactors together with one or two organic metallopterin ligands (Mo-co or W-co).^1,3,4^ While Mo is utilized by most organisms, including humans,^4,5^ W is limited to (mostly anaerobic) bacteria or archaea.^1−3,6^ The enzymes carrying Mo- or W-cofactors are represented by four unrelated families, which are named after their first structurally characterized members: sulfite oxidase (only Mo), xanthine dehydrogenase (only Mo), DMSO reductase (Mo or W), and AOR families (predominantly W). Most of these enzymes either oxidize or reduce their substrates, and some are capable of catalyzing redox reactions reversibly in both directions dependent on the respective electron donors or acceptors provided.^1,4,7^

**Figure 1:**
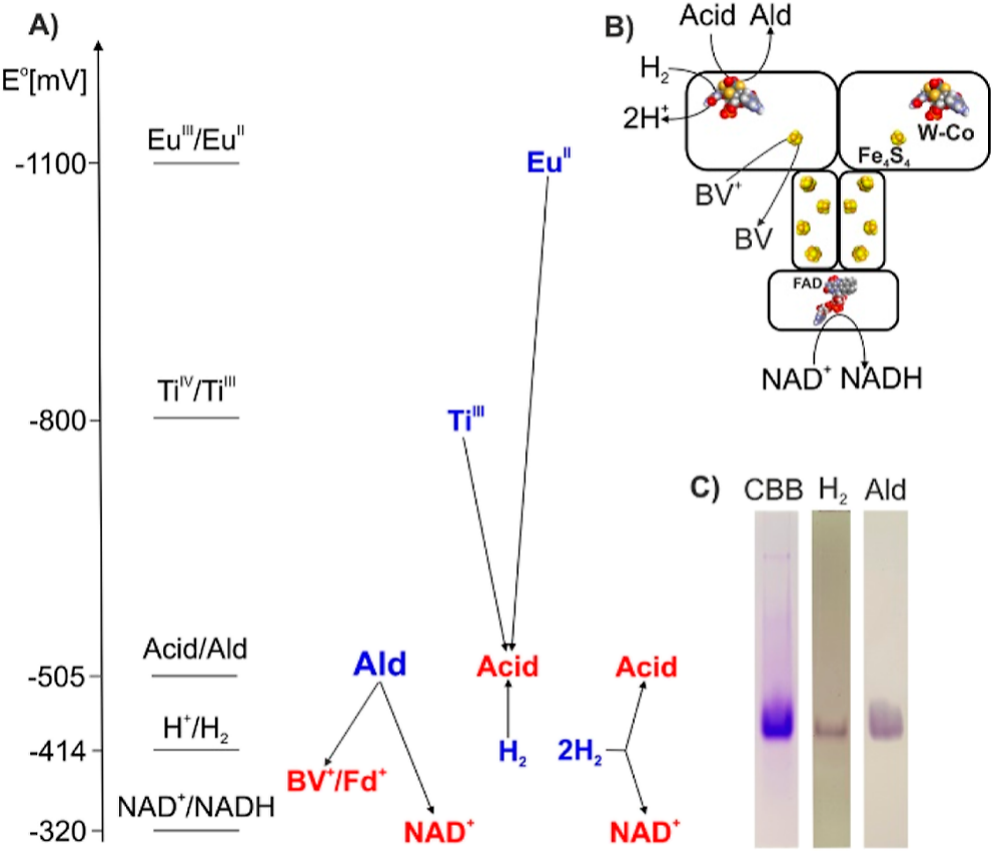
Reactions catalyzed by AOR_*Aa*_. (A) Standard redox potential scheme of the observed reactivities of AOR_*Aa*_; electron donors are shown in blue and electron acceptors in red; (B) hypothetical structure and localization of partial reactions of AOR_*Aa*_; and (C) activity staining analysis of AOR_*Aa*_ resolved by native gel electrophoresis: Coomassie stain (left), H_2_-dependent (middle) and benzaldehyde-dependent activities (right).

The W-dependent^1^ AOR constitute a family of oxygen-sensitive W-*bis*-metallopterin phosphate (MPT)-containing enzymes together with the recently discovered class II benzoyl-CoA reductases.^1^ They catalyze the oxidation of various aldehydes to the respective acids and are grouped into several branches according to their sequence conservation and their substrate preference: for example, oxidoreductases for formaldehyde (FOR),^8^ glyceraldehyde phosphate (GAPOR and GOR),^9,10^ or wide-range spectra of different aldehydes (AOR *sensu stricto*(11−16) or WOR5^17^). They are unique in biochemistry also for their ability to catalyze the thermodynamically difficult reduction of non-activated carboxylic acids to aldehydes, which usually needs prior activation to acyl phosphates or acyl thioesters.^12,15,18,19^ Activities of acid reduction have previously been demonstrated with AOR from *Thermococcus paralvinellae*(18) and *Moorella thermoacetica* (*M. thermoacetica*),^12,19^ albeit at much lower rates than that shown for aldehyde oxidation and only when very low-potential electron donors such as reduced methyl viologen or tetramethyl viologen were used as respective reductants. Because of the very low redox potentials of acid/aldehyde couples (*E*°′ < −500 mV,^15^Figure 1A), acid reduction requires such low-potential electron donors and still proceeds only at rates of less than 5% of those observed for aldehyde oxidation.^18,19^ AORs were previously thought to occur predominantly in thermophilic anaerobic microorganisms, such as the archaeal genera *Pyrococcus* or *Thermococcus* or the bacterial species *M. thermoacetica*,^20,21^ but recent findings show that these enzymes are much more widespread and also occur in many mesophilic species of strictly or facultative anaerobic Archaea and Bacteria.^1,15,16,22,23^ One of these AORs has recently been characterized from the betaprotebacterium *A. aromaticum* (AOR_*Aa*_). It occupies a separate branch in the phylogenetic tree than archaeal AOR and exhibits a more complex composition: while archaeal AOR is a homodimer of one subunit, AOR_*Aa*_ contains three subunits in (αβ)_2_γ composition, in which the β-subunits contain the W-Co and one Fe_4_S_4_ cluster, the α-subunits four more Fe_4_S_4-_clusters, and the γ-subunit a FAD cofactor.^15^ The α and β subunits comprise a two-subunit module of an AOR-type tungsten enzyme with a polyferredoxin subunit, connected with a γ subunit affiliated with FAD-containing oxidoreductases.^1,15^ While the previously known AORs are only reactive with ferredoxin or viologen dyes,^11,12,24^ AOR_*Aa*_ also accepts NAD^+^ as an additional electron acceptor for aldehyde oxidation.^15^

In this study, we investigate the recombinantly produced AOR_*Aa*_ with higher specific activity and cofactor occupancy than that obtained in the native enzyme^15^ and report on the discovery of its unexpected hydrogenase activity. We show that AOR_*Aa*_ utilizes H_2_ as an electron donor for either acid or NAD^+^ reduction, which appears to compete for the electrons provided from H_2_, rather than depend on an energy-coupled bifurcation process. We show that both reactions can be coupled with other biotechnologically useful enzyme reactions, providing the basis for a potential new system for biotechnological acid conversion or NADH recycling.

## Methods

Further information on materials and methods are presented in the Supporting Information.

### Chemicals

All organic acids (Sigma-Aldrich) used in substrate screening and activity tests were solubilized by the addition of equimolar NaOH.

### Molecular Biological and Protein Techniques

#### AOR_*Aa*_ Plasmid Construction

We used two plasmids for recombinant AOR_*Aa*_ production, containing either *aorABC* or *aorABCDE* (see the Supporting Information). The genes encoding the AOR_*Aa*_ protein from *A. aromaticum* (genes *ebA5004*-*5010*) were obtained from chromosomal DNA by PCR amplification (details given in the Supporting Information) and ligated into a mobilizable vector based on the broad-range vector pBBR.^25,26^ The obtained plasmids were transformed into a conjugation strain of *Escherichia coli* (*E. coli*) (*e.g.*, strain WM3064^27^). The final plasmid used contained the genes for the three subunits of AOR_*Aa*_ (AorABC: WP_011238651–WP_011238653) and the putative maturation factors AorD and AorE (WP_041646405 and WP_041646407) behind an anhydrotetracycline (AHT)-inducible promoter with an N-terminal Twin-Strep-tag fused to AorA. The plasmid was then transferred from the donor *E. coli* strain to *Aromatoleum evansii* (*A. evansii*) by conjugation.^26^ The recombinant *A. evansii* strain was grown anaerobically on a minimal medium (pH 7.8) with benzoate and nitrate as described in Salii *et al.*,^26^ with the addition of ampicillin and Na_2_WO_4_ to final concentrations of 100 μg/mL and 18 nM, respectively.

#### Recombinant AOR_*Aa*_ Production and Purification

Recombinant *A. evansii* cells were cultivated anaerobically either in 30 L fermenters or 2 L stoppered bottles with gentle shaking with periodic supplementation of nitrate and sodium benzoate (to concentrations of 10 and 4 mM, respectively), while monitoring nitrate and nitrite levels by test strips (Quantofix, Machery-Nagel, Düren, Germany). The cultures were inoculated with 1% (v/v) of a preculture in the same medium, and cultivation was performed at 28 °C until the optical density (OD600) reached 0.6. At this point, the temperature was set to 18 °C and enzyme production was induced by the addition of AHT to a concentration of 200 ng/mL, while the culture was also supplemented with additional Na_2_WO_4_ to a final concentration of 10 μM. After 20 h of incubation, the cells were harvested by centrifugation (4500*g*, 1 h, 4 °C) and either frozen in liquid nitrogen and stored at −80 °C or processed further.

The cells were suspended in a stock buffer [100 mM Tris/HCl pH 8.0, 150 mM sodium chloride, 10% (w/v) glycerol] with 0.1 mg/mL DNase I and 0.1 mg/mL lysozyme and subsequently lysed by ultrasonication (Sonics Vibra-Cell VCX500, intermittent cycle, 5 min, amplitude 40%, energy 150,000 J). Cell debris was separated by ultracentrifugation (40,000*g*, 1 h, 4 °C), and the cell-free extract was then applied to a Strep-tag II affinity column (IBA Lifesciences, Göttingen, Germany). After rinsing with a stock buffer, the enzyme was eluted with elution buffer consisting of stock buffer with 10 mM desthiobiotin. The enzyme thus obtained was frozen at −80 °C and stored until use.

#### Recombinant Benzyl Alcohol Dehydrogenase (BaDH) Production and Purification

The *bdh* gene of *A. aromaticum* strain EbN1 coding for a benzyl alcohol dehydrogenase (BaDH; ebA3166^28^) was used to produce BaDH *via* recombinant expression in *E. coli* BL21(DE3). Production and purification followed the protocol described in Schühle *et al.*(29) as detailed in the Supporting Information. Purified BaDH was checked for its activity by spectrophotometric assays and turned out to be specific for benzyl alcohol and only a few close chemical analogues but accepted NAD^+^ or NADP^+^ almost equally well as electron acceptors. In addition, it also catalyzed the reduction of benzaldehyde with either NADH or NADPH as an electron donor (manuscript in preparation).

### Biochemical Characterization of AOR

The metal content of enriched protein fractions or purified AOR_*Aa*_ was analyzed by inductively coupled plasma mass spectrometry (ICP-MS).^15,23^ Protein concentrations were determined by the Coomassie dye-binding assay.^30^ Standard activity assays with benzaldehyde were performed under anaerobic conditions (2% H_2_) at 30 °C in 980 μL buffer Tris/HCl pH 8.0 containing 5 μg/mL of AOR_*Aa*_ and 1.6 mM BV^2+^ or 1 mM NAD^+^ as respective electron acceptors. The reaction was initiated with the addition of 20 μL of 40 mM benzaldehyde in water.^15^ In each assay with BV^2+^, 20 μM Na_2_S_2_O_4_ was added to the buffer to reduce traces of oxygen. The obtained average specific activity was used to compare AOR_*Aa*_ batches from different purifications. We observed the same benzaldehyde oxidation rates with NAD^+^ also under aerobic assay conditions and used these to determine the apparent kinetic parameters for 0–33 mM benzaldehyde at a constant concentration of 1 mM NAD^+^. Each reaction was conducted in triplicate. Rate values were obtained from the initial phases (0–30 s) of the activity curves, and the obtained data were fitted with the Michaelis–Menten model by non-linear regression (OriginPro 2019).

#### Acid Reduction Activity of AOR_*Aa*_ with Different Electron Donors [Ti(III), Eu(II), and H_2_]

A Ti(III)-citrate stock solution was prepared as in Arndt *et al.*(15) using citric buffer pH 4.5. An Eu(II) complex stock was prepared from EuBr_2_ by dissolving the salt anaerobically in an EGTA [ethylene glycol-bis(2-aminoethylether)-*N*,*N*,*N*′,*N*′-tetraacetic acid] solution at a molar ratio of 1:1 to achieve a stock solution of 200 mM Eu(II)-EGTA.^31^ The reaction mixture contained 30 mM sodium benzoate in 100 mM citrate buffer (pH 5.5) and 0.2 μM AOR_*Aa*_ and the respective electron donor: for hydrogen as the electron donor, the mixture was equilibrated overnight in an N_2_/H_2_ 97.5%/2.5% atmosphere, whereas for the metallic electron donors, we added 3 mM Ti (III)-citrate or Eu (II)-EGTA into closed anaerobic vials under a hydrogen-free N_2_ atmosphere. The reactions were followed by the detection of benzaldehyde by HPLC method 1 (see the Supporting Information).

### Characterization of Hydrogen-Dependent NAD^+^ and BV^2+^ Reduction

In contrast to aldehyde oxidation activity, the reactivity of AOR_*Aa*_ with H_2_ depended on strictly anaerobic conditions and was inhibited even by traces of oxygen. All buffers used for reaction with H_2_ were stored in contact with a gas mixture of 2.5/97.5% H_2_/N_2_ v/v (if not stated otherwise) for >1 h to ensure proper equilibration of the gas in the liquid phase and remove traces of oxygen.

The H_2_-dependent reduction of NAD^+^ or BV^2+^ was carried out at 30 °C in 1 mL of 100 mM Tris/HCl buffer pH 8.0 containing 1 mM NAD^+^ or BV^2+^ as an electron acceptor and 10 μg/mL of AOR_*Aa*_. The reactions were started with the addition of NAD^+^ or BV^2+^ and were followed at 340 nm (ε = 6220 M^–1^ cm^–1^) or 600 nm (ε = 7400 M^–1^ cm^–1^), respectively. The pH optimum of the reaction was established with NAD^+^ as electron acceptor with 50 mM K_2_HPO_4_/KH_2_PO_4_ buffer (pH range 5.5–8.0) and 100 mM Tris/HCl (pH 7.6–8.0). The apparent kinetic parameters of NAD^+^ or BV^2+^ at pH 8.0 for reduction with H_2_ were established photometrically, either with a constant partial pressure of H_2_ [2.5% (v/v)] and varied NAD^+^ concentrations (0–2 mM) or with constant concentrations of either BV^2+^ or NAD^+^ (1 mM) and varied partial pressure of H_2_ [0–2.5% (v/v)].

The relative reactivities with NAD^+^ (at 0.1 or 1 mM) and benzoate (at 30 mM) were compared at pH 5.6 and 7.0 (100 mM citric acid/Na_2_HPO_4_ buffer), which still allowed considerable activities with both substrates. H_2_-dependent benzoate reduction was carried out by 0.02 mg/mL AOR_*Aa*_ and analyzed by HPLC method 2 (see the Supporting Information).

### Activity Staining

Purified AOR_*Aa*_ was separated by native PAGE (7% polyacrylamide in 380 mM Tris-Cl pH 8.8), and the gels were subsequently stained for aldehyde- or H_2_-dependent *in situ* NAD^+^ reduction by incubation in a 50 mM Tris/HCl buffer pH 7.0 containing 1 mM NAD^+^, 1.3 mM phenazine methosulfate, and 0.5 mM nitro blue tetrazolium chloride.^32^ The H_2_-dependent activity was observed by incubation under a protective gas atmosphere containing 3.5% hydrogen for 20–60 min, while aldehyde-dependent activity was observed *via* the addition of 1 mM benzaldehyde to the staining solution under a nitrogen atmosphere for 1–5 min.

### H_2_ Evolution Assays

Experiments concerning potential hydrogen formation by AOR were performed in 50 mM MOPS/NaOH buffer (pH 7.0) in a volume of 1.5 mL in rubber-stoppered glass bottles (*ca.* 8 mL). Each reaction mixture included AOR (0.1 mg/mL) and different electron donors [3 mM benzaldehyde, 3 mM acetaldehyde, 5 mM Ti(III)-citrate, 5 mM Eu(II)-EGTA, 5 mM NADH, or 1 mM NADH with additional 5 mM sodium formate and formate dehydrogenase]. Prior to mixing, all components were flushed with nitrogen gas to remove traces of dissolved hydrogen from the residual anaerobic chamber atmosphere. Bottles were incubated upside down at room temperature for 3 h. Afterward, the gas phases were analyzed *via* GC-TCD together with standard gas mixes containing 0.5–4.0% H_2_ in N_2_ (v/v). No production of H_2_ above the detection limit (0.1% H_2_) was observed with any of the tested electron donors.

### Coupled Enzyme Assays

The apparent kinetic parameters for the reduction of benzoic acid with H_2_ were determined in a coupled assay with added BaDH, which was converting the produced benzaldehyde to benzyl alcohol in an NADPH-coupled reduction. The change in NADPH concentration was followed at 365 nm (ε = 3.5 mM^–1^ cm^–1^) and benzaldehyde and benzyl alcohol production was quantitated by HPLC method 2 (see the Supporting Information). The assay was performed under 2.5% hydrogen in nitrogen (v/v) in the headspace at 30 °C. The reaction mixture contained 100 mM citric buffer pH 5.5, 0.6 mM NADPH, BaDH (170 μg/mL), and AOR_*Aa*_ (0.03 mg/mL). The reaction was initiated by the addition of sodium benzoate to concentrations of 4–42 mM in the reaction mixture. The pH optimum of AOR_*Aa*_ for benzoate reduction with H_2_ (2.5% v/v) was determined with the same coupled enzyme assay using 30 mM sodium benzoate and 100 mM citric buffer in the pH range of 4.5–6.0 and 50 mM K_2_HPO_4_/KH_2_PO_4_ buffer in the pH range of 6.0–7.0.

The cascade reactions with NAD^+^ instead of NADPH were conducted under an anaerobic atmosphere at 30 °C and a pH of either 5.6 or 7.0 (100 mM citric acid/Na_2_HPO_4_ buffer) in a 2 mL cuvette containing 0.1 mM NAD^+^, 30 mM sodium benzoate, 20 μg/mL AOR_*Aa*_, and 75 μg/mL BaDH. The concentration of NADH was followed spectrophotometrically at 340 nm, while benzaldehyde and benzyl alcohol concentrations were followed by HPLC method 2.

### Substrate Specificity

The reactivity of AOR_*Aa*_ with benzoate, 4-hydroxybenzoate, phenylacetate, *trans*-cinnamate, nicotinate, and octanoate was proved by the detection of the corresponding aldehydes in the reaction mixture by LC–ESI(+)-MS/MS for 4-hydroxybenzaldehyde or by GC-MS for the other aldehydes (details given in the Supporting Information). The reactions were performed anaerobically in stoppered vials and flushed with pure hydrogen for 5 min, and the reaction mixture contained 50 mM MES buffer pH 5.5 and 0.1 μM AOR_*Aa*_. The reaction mixture was mixed gently for 3 h at 25 °C. The methods for stopping the reaction, sample preparation, and identification of the respective aldehyde are described in the Supporting Information.

### Isotope Tests with D_2_O/H_2_O and H_2_/D_2_

The reaction of carboxylic acid reduction was performed anaerobically in stoppered vials and flushed with pure hydrogen (or deuterium) for 5 min. The reaction volume was 10 mL in 50 mM MES buffer pH 6.5. The concentration of sodium benzoate was 20 mM, and the enzyme concentration was 0.1 μM. For the reaction in D_2_O, a buffer was prepared as a 50x stock, which resulted (including an added enzyme) in a final concentration of 95% D_2_O/5% H_2_O in the assays. Sample preparation and benzaldehyde detection with GC-MS were conducted as described for the substrate screening assays.

### Reactor for Reduction of Acetophenone to (*R*)-1-Phenylethanol

Cell extract containing recombinant AOR_*Aa*_ was desalted by repeated 5-fold concentration *via* ultrafiltration (10 kDa membrane, Millipore Amicon Ultra-15 mL) and subsequent dilution with 100 mM Tris/HCl buffer pH 8.0. The extract concentration for the assays was adjusted to 1 mg/mL protein, and after adding 0.5 mM NAD^+^, 2 mM acetophenone, and 6 ng/mL of an (*R*)-specific 1-phenylethanol dehydrogenase (R-HPED^33−35^), the mixture was exposed to 2% H_2_ under anaerobic conditions. The samples were taken at several time points in duplicate, the reaction was stopped by mixing with acetonitrile in a 1:1 (v/v) ratio, and the produced (*R*)-1-phenylethanol was detected by the RP-HPLC method (see the Supporting Information).

## Results

### Biosynthesis and Purification of Recombinant AOR_Aa_

We have established a recombinant expression system for AOR_*Aa*_ from *A. aromaticum* using the related species *A. evansii*(36) as a host organism, which is equipped for synthesizing and incorporating the tungsten cofactor. The plasmid contains the *aorABC* genes encoding the three subunits of AOR_*Aa*_^15^ and two additional genes for potential cofactor maturation factors, *aorD* and *aorE* (Figure S1 of the Supporting Information). To facilitate purification, a Twin-Strep-tag was fused to the N-terminus of AorA. The addition of the *aorDE* genes into the expression vector as well as supplying the culture with relatively high tungstate concentrations (10 μM) after induction of gene expression resulted in significant improvements of specific activity and W-content of purified AOR_*Aa*_, as indicated in Table 1. The specific activity of recombinant AOR_*Aa*_ was 3.6-fold higher than that of native AOR_*Aa*_,^15^ and it showed an almost full occupation of tungsten, as indicated by elemental analysis (1.52 mol/mol W of 2.0 expected) (Figure 1B and Table 1). The obtained Mg (bridging the two metallopterins) and P contents indicate that the metallopterin and FAD cofactors are also present at full occupancy, while the Fe-S clusters may be only partially occupied, judging from the recorded Fe content (Table 1). From these results, we assume that recombinant AOR_*Aa*_ represents a fully functional enzyme for further analysis.

**Table 1:** Dependence of Specific Benzaldehyde Oxidation Activities Using BV^2+^ as an Electron Acceptor and Elemental Contents of Recombinant AOR_*Aa*_ on the Presence of *aorDE* and Tungstate Supplementation (+W)

		elemental content			
enzyme type	SA [U/mg]	W/Mo	Fe	Mg	P
aor_ABC	4.5	1.06/0	36.2	1.43	5.22
aor_ABCDE	16	0.39/0	32.6	1.10	8.93
aor_ABCDE + W	85	1.52/0	23.1	1.79	9.93
native AOR^a^	23.6	1.46/0	32.1	1.92	5.12

a Data shown previously in Arndt *et al*.^15^

### Acid Reduction Activity of AOR_*Aa*_ with Different Electron Donors [Ti(III), Eu(II), and H_2_]

The principal ability of AOR_*Aa*_ to catalyze the reduction of benzoate to benzaldehyde with titanium (III) citrate as an electron donor was shown in our previous study by the identification of a 3-nitrophenylhydrazone derivative of the benzaldehyde.^15^ We have now developed a quantitative method of benzaldehyde detection and identified and compared the reaction with different low-potential electron donors. Purified AOR_*Aa*_ reduced benzoate under thermodynamically favorable conditions, that is, using a pH of 5.5, 30 mM benzoate, and 3 mM of low-potential electron donors [either titanium (III)-citrate or europium (II)-EGTA complexes)], yielding considerable amounts of product (11 or 33 μM benzaldehyde, respectively). Surprisingly, when AOR_*Aa*_ was exposed to hydrogen as an electron donor (2.5% partial pressure, pH 5.5), it also reduced benzoate and reached the same yield (11 μM) as with Ti(III)-citrate, despite the higher standard redox potential of hydrogen [*E*°′(H^+^/H_2_) = −414 mV, *E*°′(benzoic acid/benzaldehyde) = −505 mV, as calculated from Δ*G*°_f_ values from,^37^ see Figure 1A]. Although some Mo-enzymes have previously been reported to react with hydrogen as an electron donor,^32,38,39^ AOR_*Aa*_ exhibited rates of reactivity never observed before with any tungsten or molybdenum enzyme. The ability of AOR_*Aa*_ for hydrogen-dependent NAD^+^ reduction was also evident from activity staining assays after native polyacrylamide electrophoresis of the purified enzyme, which showed equally strong staining with hydrogen as an electron donor, but at a slower rate than with aldehydes (Figure 1C). Therefore, we investigated this novel feature more extensively.

### Hydrogenase Activity of AOR_*Aa*_

We confirmed that hydrogen serves as an electron donor for all previously described reactivities of purified AOR_*Aa*_, namely, reduction of acids, benzyl viologen, or NAD^+^. The pH optimum for NAD^+^ reduction was observed at pH 8.0 (Figure S2), similar to the pH optimum for oxidation of aldehydes,^15^ whereas acid reduction reaction was 7 times faster at acidic (pH 5.6) than at neutral conditions (pH 7.0) (Figures S3A and S5A and Table 2). To characterize the new AOR reactivity of reducing NAD^+^ or BV^2+^ with molecular hydrogen in more detail, we analyzed the apparent kinetic parameters at the optimum pH of 8.0 in a steady-state kinetics study (Table 3 and Figure S6). The assays were performed either with varied hydrogen concentrations (0–2.7%) and constant NAD^+^ or BV^2+^ concentrations (1 mM), or at a constant hydrogen concentration (2.5%) and varied concentrations of NAD^+^ (0–2 mM). The enzyme showed apparent *k*_cat_ values of 10.2 s^–1^ (or 13.4 s^–1^ for varied NAD^+^) for H_2_ oxidation coupled to the reduction of NAD^+^, and 17.9 s^–1^ coupled to BV^2+^ reduction, which is consistent with the previously observed differences in aldehyde-dependent reduction with these electron acceptors.^15^ In the case of benzaldehyde-dependent NAD^+^ reduction (pH 8.0), AOR_*Aa*_ exhibited a high affinity for benzaldehyde with a *K*_m_ of 39 μM and an approximately 10-fold higher reaction rate (*k*_cat_ 123 s^–1^) than with hydrogen. Although the calculated *k*_cat_ values for the experiments with H_2_-dependent NAD^+^ reduction represent only 8.3–10.5% of the those observed for benzaldehyde-dependent NAD^+^ reduction, the very low apparent *K*_m_ values of either H_2_ or NAD^+^ (0.4% of H_2_ in the headspace or 0.3 μM of dissolved H_2_ and 21.7 μM NAD^+^, respectively) indicate that the reactivity of AOR_*Aa*_ with H_2_ is physiologically relevant. The observed turnover rates for hydrogen-dependent NAD^+^ reduction represent the highest hydrogen oxidation rate of any Mo-or W-enzyme to date.^38^ Steady-state kinetics of benzoate reduction with H_2_ was investigated by a coupled assay with benzyl alcohol dehydrogenase (BaDH) because enzyme-free controls showed that benzaldehyde is unstable and gets degraded over the recorded time period, and the recorded initial concentrations were close to the HPLC detection limit. Interestingly, the observed benzoate reduction progress curves apparently begin with a burst phase (1–5 min) reaching benzaldehyde concentrations of 5–25 μM (depending on pH and experimental conditions, see Figures S3 and S5), followed by a further linear increase (Figure S5). In experiments with higher enzyme concentrations, we observed a fast accumulation of benzaldehyde to steady-state concentrations in the range of 100–150 μM (Figure S3G,H). We also assayed for potential hydrogen production by AOR_*Aa*_ using various possible electron donors (see Methods) but never observed any H_2_ production in the gas phase above the detection limit.

**Table 2:** Steady-State Activities of AOR_*Aa*_ for NAD^+^ or Benzoate Reduction with Hydrogen as the Electron Donor^a^

		specific activity [mU/mg]	
reaction set-up	data for substrate	pH 5.6	pH 7.0
benzoate	acid	74	10
0.1 mM NAD^+^	NAD^+^	16	1.3 × 10^3^
1 mM NAD^+^	NAD^+^	72	1.6 × 10^3^
0.1 mM NAD^+^ + benzoate	acid	52	14
	NAD^+^	7.3	1.0 × 10^3^
1 mM NAD^+^ + benzoate	acid	6	n.d.
	NAD^+^	49	1.7 × 10^3^
1 mM benzaldehyde	aldehyde	805	1.4 × 10^4^

a Reactions contained either 30 mM sodium benzoate, 0.1 mM or 1 mM NAD^+^ as electron acceptors, or combinations thereof at pH 5.6 and 7.0, respectively. Oxidation of 1 mM benzaldehyde with 1 mM NAD^+^ is shown as a control.

**Table 3:** Apparent Kinetic Parameters of AOR_*Aa*_; *k*_cat_ was Calculated for the Mass of the Complex (αβ)_2_γ of 218 kDa

[A]	[B]_const_	*V*_max_ [U/mg]	*K*_M_ [μM] or [%]^a^	*k*_cat_ [s^–1^]	*k*_cat_/*K*_M_ [μM^–1^ s^–1^]
NAD^+^	H_2_	3.7 ± 0.1	21.7 ± 2.7	13.4	0.6
H_2_	BV^2+^	4.94 ± 0.26	2.8 ± 0.6	17.9	6.4
			[0.38 ± 0.08]^a^		
H_2_	NAD^+^	2.82 ±0.19	0.3 ± 0.3	10.2	34.2
			[0.037 ± 0.034]^a^		
benzoate	H_2_	0.39± 0.03	33.700 ± 566	1.4	4.2 × 10^–5^
benzaldehyde	NAD^+^	33.8 ± 1.2	39.3 ± 6.6	123	3.1

a Hydrogen concentration in μM was calculated from H_2_ pp % according to its solubility coefficient in water at 30 °C and 1 atm (*q* = 0.0001474 g hydrogen in 100 g of water).

The reactivity of AOR_*Aa*_ in reducing either BV^2+^ or NAD^+^ with H_2_ as an electron donor raises the question of whether this represents a new example of electron bifurcation since the standard redox potential of H_2_ (−420 mV) is approximately in the middle between those of the benzoate/benzaldehyde (−505 mV) and NADH/NAD^+^ couples (−320 mV) (Figure 1A). Therefore, we set up experiments monitoring the H_2_-dependent reduction of benzoate and NAD^+^ simultaneously by spectrophotometrically following NADH production and quantitating benzaldehyde production by HPLC.

We compared the time-dependent activities of AOR_*Aa*_ at pH 5.6 and 7.0 for simultaneous reduction of benzoate (30 mM) and NAD^+^ (1.0 or 0.1 mM) (Tables 2 and S1). These pH values represent a compromise to approach the respective optima for acid or NAD^+^ reduction while still retaining the significant activity of the other reaction. The study revealed that rates of acid and NAD^+^ reduction at pH 5.6 depended on NAD^+^ concentration, showing an 8-fold slower reduction of the acid than NAD^+^ at 1 mM NAD^+^ (6 mU/mg *vs* 49.3 mU/mg), whereas this ratio was reversed at 0.1 mM NAD^+^ (52.4 mU/mg for benzoate and 7.3 mU/mg for NAD^+^ reduction, Figures S2 and S3). Thus, benzoate reduction rates apparently decrease with increasing content of NAD^+^ (activity without added NAD^+^: 74 mU/mg, Figure S3A,B).

At pH 7.0, we observed a much faster NAD^+^ reduction rate (1013 mU/mg), leading to fast NAD^+^ depletion in the experiments with 0.1 mM NAD^+^ (Figure S4). Therefore, the much slower acid reduction was not affected in the experiment with 0.1 mM NAD^+^ and occurred at approximately the same rates as with benzoate alone (14 and 10 mU/mg, respectively). In similar experiments with elevated NAD^+^ concentration (1 mM), NAD^+^ was not depleted during the observed time frame, resulting in the almost complete shutdown of acid reduction (Figures S3 and S4).

These data indicate that the observed simultaneous reduction processes of acid and NAD^+^ are neither synergetic nor stoichiometrically coupled, as would be expected from an electron bifurcating process. Therefore, the observed effects do not qualify as electron bifurcation and rather indicate competition for electrons between these two electron acceptors.

### Reactivity of AOR_*Aa*_ with Other Acids

In addition to benzoate, several other acids were converted to the corresponding aldehydes by AOR_*Aa*_ in the same hydrogen-dependent reduction process based on the identification of the products by GC–MS or LC–MS methods. As in the case of aldehyde oxidation, AOR_*Aa*_ showed a very broad substrate range including aromatic and alkylaromatic (benzoic acid, 4-hydroxybenzoic acid, vanillic acid, phenylacetic acid, and *trans*-cinnamic acid), heterocyclic (nicotinic acid), and aliphatic (octanoic acid) acids. We observed M^+^ MS signals and characteristic aldehyde fragmentation patterns in GC–MS as well as [M + H]^+^ quasi-molecular parent ions and characteristic fragmentations in LC–MS/MS (Table S2). The identity of the aldehyde products was additionally confirmed by applying standard compounds.

### Cascade Reactions

**Figure 2:**
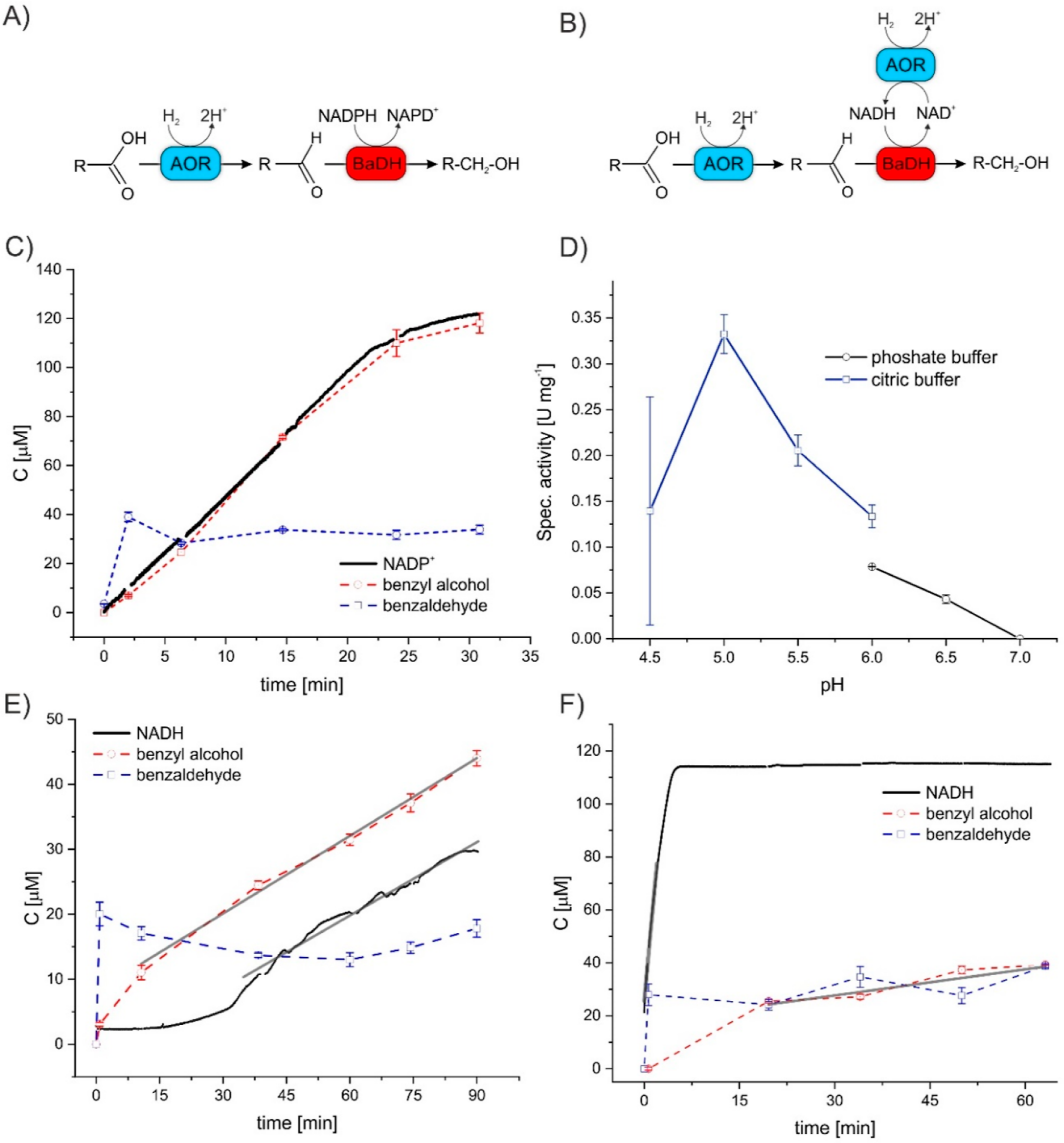
Cascade reactions of reduction of benzoate to benzaldehyde by AOR_*Aa*_ followed by a further reduction to benzyl alcohol by BaDH. (A) NADPH-dependent and (B) NAD^+^-dependent reaction setup, (C) progress curve of the coupled assay with NADPH at pH 5.5, (D) pH dependence of the coupled assay with NADPH, measured in citric buffer (blue line) or phosphate buffer (black line), and (E) progress curve of the coupled assay with NAD^+^ at pH of 5.6 and (F) at pH 7.0; blue dashed lines/squares represent benzaldehyde, red dashed lines/circles represent benzyl alcohol, black lines represent NADH/NADPH concentrations, and gray lines show fitted linear trends. Error bars represent standard deviations.

To avoid probable feedback inhibition effects by the produced benzaldehyde on AOR_*Aa*_ activity in benzoate reduction and prevent interference from benzaldehyde instability over extended incubation times (as observed in enzyme-free controls), we developed coupled assays with a benzyl alcohol dehydrogenase (BaDH) of *A. aromaticum* (protein accession no. WP_011237618), in which benzaldehyde was immediately removed from the equilibrium by reducing it further to benzyl alcohol. BaDH specifically interconverts benzyl alcohol and benzaldehyde and a few substituted derivatives and accepts either NAD^+^ or NADP^+^ as redox coenzymes, in contrast to AOR_*Aa*_, which only reduces NAD^+^, but not NADP^+^.^15^ Because of these different cofactor specificities, the two different nicotinamide nucleotides allowed us to distinguish between a situation where AOR_*Aa*_ only contributes benzaldehyde to the coupled reaction (using NADPH) and a situation where AOR_*Aa*_ also recycles NADH for aldehyde reduction by BaDH. Thus, H_2_-dependent benzoate reduction *via* AOR_*Aa*_ was monitored by the further reduction of benzaldehyde to benzyl alcohol with added NADPH (Figure 2A). Likewise, the simultaneous reduction of benzoate and NAD^+^ by AOR_*Aa*_ can be monitored using a coupled assay with added BaDH and NAD^+^. Under these conditions, AOR_*Aa*_ provides both benzaldehyde and NADH for benzaldehyde reduction *via* BaDH, which can be monitored by analyzing the increase of benzyl alcohol (*via* HPLC) and NADH (spectrophotometrically) (Figure 2B)

The non-impeded rate of acid reduction with hydrogen by AOR_*Aa*_ as measured in a coupled assay with BaDH and NADPH showed a linear progression at a rate of 244 mU mg^–1^ up to 100 μM of the product (measured photometrically as a decrease of NADPH absorption, Figure 2C). The pH optimum for benzoate reduction based on this coupled assay was 5.0, confirming the previous determination from non-coupled assays (Figure 2D). Under the assay conditions, the initial rate of benzaldehyde production exceeded that for benzyl alcohol and quickly entered a quasi-equilibrium phase (depending on the amount of enzyme) while the alcohol accumulated at a slower but steady rate. The rates of NADPH consumption and alcohol production were identical, confirming that NADPH was only turned over by BaDH, not by AOR_*Aa*_ (Figure 2C). This coupled test allowed us to analyze some features of AOR_*Aa*_ that were not accessible otherwise. We have used this test to analyze the kinetics of H_2_-dependent acid reduction, revealing apparent *K*_m_ and *k*_cat_ values for benzoate reduction (33.7 mM benzoate and 1.4 s^–1^, respectively), as measured at 2.5% H_2_ concentration and pH 5.5.

In addition, we observed apparent substrate inhibition on benzoate reduction by H_2_ concentrations higher than 2.5%: the rate of benzoate reduction dropped to 60% of the maximum with 20% H_2_ and to 40% with 40% H_2_. Notably, no such substrate inhibition was observed for H_2_-dependent NAD^+^ reduction.

The second coupled test containing BaDH and NAD^+^ was intended to analyze the reaction stoichiometry and to test the potential technological applicability for reducing non-activated acids to the corresponding alcohols. Using H_2_ as an electron donor, AOR_*Aa*_ was expected to catalyze both the reduction of benzoate to benzaldehyde and of NAD^+^ to NADH, which would be recycled by BaDH during the further reduction of benzaldehyde to benzyl alcohol (Figure 2B). Therefore, in an optimally coupled reaction cycle, we would expect an H_2_-dependent reduction of benzoate to benzyl alcohol without accumulation of either benzaldehyde or NADH. The experiment as conducted at acidic pH (5.6, Figures 2E and S7A,B) showed a quick establishment of a quasi-equilibrium concentration of aldehyde at approx. 20 μM and a linear increase of alcohol concentrations after 15 min at the rate of 20 mU^.^mg^–1^. During the first 30 min of the reaction, we observed almost no accumulation of NADH due to its consumption to reduce the aldehyde. However, at a later stage, we observed an increase of NADH concentration at the same rate as for alcohol (19 mU mg^–1^).

Meanwhile, at neutral pH, we observed a high rate of NADH production (1300 mU mg^–1^), whereas the formation of benzaldehyde was again followed by its reduction to alcohol at a constant rate of 17 mU mg^–1^ after 15 min (Figures 2F and S7C,D), similar to the benzyl alcohol production rate at pH 5.6. Therefore, the coupled enzyme system appears to be equally applicable at highly divergent pH settings in contrast to acid reduction with just AOR_*Aa*_.

### D_2_/D_2_O Experiments

To gain additional insights into the mechanism of benzoate catalytic reduction with H_2_, we have analyzed the isotope pattern of the produced benzaldehyde by recording the mass spectra after reaction with (i) D_2_ in H_2_O, (ii) H_2_ in D_2_O, and (iii) H_2_ and Eu(II) complex in D_2_O and as a control (iv) with H_2_ in H_2_O (see Table S3). The fraction of deuterated benzaldehyde was estimated based on the intensity of the 108 *m*/*z* signal relative to its occurrence in deuterated or non-deuterated benzaldehyde standards {[M + 1]^+^ and [M + 2]^+^, respectively}.

In samples reduced with D_2_ in H_2_O, we observed only a small (8.4%) enrichment of benzaldehyde with deuterium. However, much higher contents of deuterated benzaldehyde were obtained when the reduction was conducted in D_2_O with H_2_, that is, 27 and 76.7%, respectively, for H_2_/D_2_O and H_2_/Eu/D_2_O conditions. These results suggest that protons from H_2_/D_2_ are released upon reduction of the W-cofactor and exchanged with water molecules present inside the active site. Therefore, the proton required for benzaldehyde formation does not originate from a direct hydride transfer from H_2_/D_2_*via* the W-*bis*-MPT cofactor to the carbonyl atom of benzoate but from the solvent or proton donors exchanging with the solvent.

### Potential Applications

To demonstrate the biotechnological potential of AOR_*Aa*_, we have conducted a synthesis of benzyl alcohol from benzoate with an AOR_*Aa*_/BaDH cascade system and NAD^+^ regeneration. The reaction was set up with 0.1 mM NAD^+^, 30 mM benzoate, and 3% H_2_ in the headspace (v/v) at pH 5.5, and after 160 min of reaction, 0.215 mM of benzyl alcohol was obtained. The initial rate of the reaction was slowed due to the low concentration of NADH, which was being produced by AOR_*Aa*_ (Figure S8A). Furthermore, the potential application of AOR_*Aa*_ for H_2_-dependent NADH recycling was demonstrated in a reactor containing a short-chain (*R*)-1-phenylethanol dehydrogenase from *A. aromaticum* (R-HPED), which was used to reduce acetophenone to (*R*)-1-phenylethanol.^33−35^ The reaction was conducted at pH 8.0 with crude cell extract as a catalyst (Figure S8B). It proceeded at a linear rate for 2 days yielding 0.43 mM of the product. Finally, AOR_*Aa*_ was tested for NAD^+^ reduction with a model syngas mixture (59% N_2_, 40% CO, and 1% H_2_). After 1.5 h of incubation in the presence of the model syngas, using crude cell extract with recombinant enzyme, the same activity was recorded as under a N_2_/H_2_ atmosphere. This demonstrates that AOR_*Aa*_ can be efficiently used even with blue hydrogen (*i.e.*, from the steam methane reforming process).

## Discussion

We report on the recombinant production of the tungsten enzyme AOR_*Aa*_ with very high specific activity and investigate its significant hydrogenase side activity, leading to the hydrogen-dependent reduction of either organic acids, BV^2+^ or NAD^+^. The production of recombinant AOR_*Aa*_ was optimized in *A. evansii* to produce an enzyme with approximately the same W-content and 3.6-fold higher specific activity compared to the purified native enzyme.^15^ AOR_*Aa*_ was shown to use low-potential Eu(II) or Ti(III) complexes as efficient electron donors for the reduction of benzoate to benzaldehyde, expanding the previous findings by Arndt *et al.*(15) Surprisingly, we also observed the reduction of acids, benzyl viologen, or NAD^+^ occurring with H_2_ as the sole electron donor, despite its apparent unsuitable redox potential for acid reduction [*E*°′(H^+^/H_2_) = −414 mV, *E*°′(benzoic acid/benzaldehyde) = −505 mV, as calculated from Δ*G*°_f_ values from.^37^ Therefore, AOR_*Aa*_ contains a previously unknown hydrogenase side activity, which is most likely attributed to the tungstopterin-active site. The specific activity of H_2_-dependent NAD^+^ reduction represents about 10% of the aldehyde-dependent rate (Table 3), exceeding any previously observed H_2_-dependent reactivities of other Mo- or W-enzymes. The highest reported rates of H_2_-dependent reduction of a Mo-enzyme were observed for CO dehydrogenase, where about 5% of the CO-dependent rates (*i.e.*, 5.1 s^–1^) were reported for the ultimate reduction of quinones or methylene blue.^38^ Interestingly, both enzymes only appear to use H_2_ as a donor but not to produce H_2_ from protons, although we tried several thermodynamically feasible electron donors for this process [*e.g.*, Eu(II), Ti(III), and aldehydes]. Other examples of hydrogenases different from the conventional NiFe-, FeFe-, or Fe-only enzymes^40,41^ are the hydrogenase side activities of nitrogenases^42^ and alkaline phosphatases reacting with phosphites.^43^ Remarkably, these alternative hydrogenase activities are limited to hydrogen evolution, whereas those of molybdenum and tungsten enzymes appear to be limited to H_2_ oxidation.

AOR_*Aa*_ actually exhibits a very high affinity for H_2_ as an electron donor, attaining half-maximal activity at approximately 0.3 μM (0.4% of H_2_ in the headspace). The observed hydrogenase activities were measured with highly purified AOR_*Aa*_, indicating the absence of potentially contaminating conventional hydrogenases. Such contaminations can also be reliably excluded because the genomes of either *A. aromaticum* or *A. evansii* do not contain any genes coding for such hydrogenases.^28^ Moreover, in contrast to conventional hydrogenases, AOR_*Aa*_ is not inhibited by CO and therefore may be applicable for exploiting H_2_ sources with high CO admixtures, for example, syngas.

Thermodynamic calculations indicate that the H_2_-dependent reduction of either NAD^+^ or BV^2+^ is already exergonic under standard conditions (Δ*G*°′ = −18.1 kJ/mol and −7.7 kJ/mol, respectively), whereas H_2_-dependent benzoate reduction is endergonic (Δ*G*°′ = +17.5 kJ/mol).^37^ However, the reaction conditions contained about 250-fold higher benzoate concentrations than benzaldehyde in steady state with a constant H_2_ concentration, resulting in a calculated Gibbs free enthalpy difference much closer to the thermodynamic equilibrium (Δ*G*′ = Δ*G*°′ + RT ln *K* = +3.8 kJ/mol with *K* = 4 x 10^–3^). Therefore, regarding some uncertainty of the available thermodynamic parameter tables, the observed reactivity of AOR_*Aa*_ is within the physicochemical expectations.

The observed H_2_-dependent benzoate and NAD^+^ reduction reactions were characterized in more detail. Benzoate reduction to benzaldehyde was dependent on a very high substrate concentration (>30 mM) and was faster at slightly acidic pH (optimum pH 5.0), suggesting that benzoic acid rather than benzoate is probably the actual substrate in the active site as proposed previously,^44^ whereas NAD^+^ reduction was recorded in a concentration range of 0.1–2.0 mM NAD^+^ and showed its optimum pH at 8.0. Because the activities with the respective alternative electron acceptor were already too low to measure at the optimum pH values, we compared the effects on simultaneous benzoate and NAD^+^ reduction at pH 5.6 and 7.0, respectively. H_2_-dependent benzoate reduction was 7-fold faster at pH 5.6 than at pH 7.0 and showed an apparent burst kinetics type with a very fast initial phase, followed by a slower linear rate, until the reaction went into a saturation phase when 100–150 μM benzaldehyde was produced. Because the recorded saturation concentrations of benzaldehyde are close to the expected values for thermodynamic equilibrium, we assume that the rates of benzoate reduction and benzaldehyde re-oxidation cancel each other out in the saturation phase. In contrast, NAD^+^ reduction always followed a linear rate until it slowed down because of NAD^+^ depletion. Moreover, the observed rates of benzoate and NAD^+^ reduction were within 1 order of magnitude in the experiments at pH 5.6 but showed a much higher difference at pH 7.0, where NAD^+^ reduction was 20- to 80-fold faster, and benzoate reduction was at least 2-fold slower than at pH 5.6.

Because the redox potential of H_2_ is about halfway between those of benzoate and NAD^+^ reduction, it is tempting to speculate about a potential electron bifurcation mechanism coupling both processes. Electron bifurcation has recently been discovered in many anaerobic microbial pathways, such as butyric acid fermentation, acetogenesis, or methanogenesis.^45−49^ It usually includes a complex redox enzyme that couples the oxidation of an electron donor of intermediate redox potential with an electron acceptor of higher potential (exergonic reaction) and a second one of lower potential (endergonic reaction), resulting in an energy-neutral overall reaction.^45,47,49^ Most of the known electron-bifurcating enzymes contain a subunit with a special flavin (or quinone) cofactor, which is able to spilt electron pairs into single electrons for transfer to the respective acceptors.^47,49^ AOR_*Aa*_ does not fit well to these requirements because the only possible cofactor candidates for this function are the W-cofactor in the β- and the FAD in the γ-subunit, whose expected functions are the partial reactions with either acids (W-cofactor) or with NAD^+^ (FAD). Furthermore, our data indicate that the observed simultaneous reduction processes of acids and NAD^+^ are neither synergetic nor stoichiometrically coupled, as would be expected from an electron bifurcating process. Therefore, the observed effects do not qualify as electron bifurcation and rather indicate competition for electrons between these two electron acceptors. Hence, AOR_*Aa*_ apparently does not belong to the growing list of anaerobic enzymes exhibiting electron bifurcation. A recent report on another AOR from an anaerobic gut bacterium actually claims a different type of electron bifurcation process but is lacking sufficient control experiments on the synergy and stoichiometry of the reactions.^16^

Acid reduction experiments with either D_2_ or D_2_O indicate that the mechanism of H_2_-dependent reduction of benzoate proceeds without directly incorporating a proton from H_2_ into the product, as indicated by the higher D incorporation rates into the generated aldehyde in experiments using D_2_O rather than those using D_2_. This indicates that the protons of H_2_ (or D_2_) are completely dissociated after reducing the W-cofactor from the W(VI) to the W(IV) state, and aldehyde formation is accompanied by taking up a proton from the available pool at the active site. This apparent proton release at the reduced W-cofactor also excludes the presence of a bound hydride equivalent at the tungsten cofactor, consistent with the observed lack of H_2_ evolution activity. The relatively low observed aldehyde deuteration rates even in the experiments in D_2_O-based buffers may be explained by the carryover of unlabeled water (or other protons) into the active site of AOR_*Aa*_.

H_2_-dependent reduction of organic acids was not only observed with benzoate, but we also showed the reduction of several other aromatic, heteroaromatic, and aliphatic acids by AOR_*Aa*_. This correlates with the very broad substrate range reported for either BV- or NAD-dependent oxidation of the respective aldehydes by AOR_*Aa*_.^15^ Therefore, AOR_*Aa*_ is applicable for converting many different organic acids into biotechnologically interesting products (*e.g.*, vanillin^50^).

To avoid the observed problems of obtaining steady-state situations after a very fast initial acid reduction, we devised a coupled benzoate reduction by AOR_*Aa*_ with BaDH from *A. aromaticum*, which reduces benzaldehyde further to benzyl alcohol using either NADPH or NADH. Because AOR_*Aa*_ only reduces NAD^+^, but not NADP^+^, the use of the two different nicotinamide nucleotides allowed us to distinguish between a situation where AOR_*Aa*_ only contributes benzaldehyde to the coupled reaction (using NADPH), or a situation where AOR_*Aa*_ also recycles NADH for aldehyde reduction by BaDH. We indeed observed the expected behavior in coupled assays with NADPH, where AOR_*Aa*_ produced a low quasi-steady-state concentration of benzaldehyde very fast, but a linear increase of benzyl alcohol accompanied by stoichiometric oxidation of NADPH was observed. Experiments with added NAD^+^ showed an overstoichiometric reduction of NAD^+^ over benzoate by AOR_*Aa*_, which became even more evident at pH 7.0, where almost all electrons were consumed by NAD^+^ reduction and only very little benzyl alcohol production was recorded. These experiments show that the acid reduction activity of AOR_*Aa*_ can principally be exploited to synthesize compounds from organic acids but also reinforce our notion that it does not use an electron bifurcation mechanism. The potential use of AOR_*Aa*_ for H_2_-dependent NADH recycling was also demonstrated in a second coupled system for producing chiral alcohols from ketones *via* a stereospecific short-chain alcohol dehydrogenase.

The coupled assays with BaDH were also used to measure the enzyme kinetic parameters of AOR_*Aa*_ with benzoate. The apparent *K*_m_ value demonstrated an 850-fold lower affinity of the enzyme for benzoate than for benzaldehyde and an 80-fold lower reduction rate than for oxidation of the benzaldehyde.

## Data Availability

The data sets generated during and analyzed during the current study are available from the corresponding author on reasonable request.

## Supporting Information Available

Extended experimental procedure (chemicals, plasmid construction, enzyme production and purification for AOR_*Aa*_ and BaDH, coupled enzyme assay, steady-state assays for AOR_*Aa*_, control experiments, HPLC, LC–MS/MS, and GC–MS); plasmid map of pAOR_1; pH dependency of AOR_*Aa*_ of NAD^+^ reduction with H_2_; kinetic progress curves and specific activities for the reduction of benzoic acid to benzaldehyde, cascade reactions, and NADH-recycled system for R-HPED; Michaelis–Menten kinetic curves; and LC–MS/MS and GC–MS results for various acids and isotope experiments (PDF)
