# Supplementary material for: Tungsten Enzyme Using Hydrogen as an Electron Donor to Reduce Carboxylic Acids and NAD+
Source: ACS Catal. 2022 Jul 6;12(14):8707–17. doi: 10.1021/acscatal.2c02147 (PMC9295118; doi:10.1021/acscatal.2c02147)
Supplement: Supplementary file 1 — cs2c02147_si_001.pdf [file cs2c02147_si_001.pdf]

SUPPORTING INFORMATION

A tungsten enzyme using hydrogen as an electron donor to reduce carboxylic acids and NAD^+

Agnieszka Winiarska[1], Dominik Hege[2], Yvonne Gemmecker[2], Joanna Kryściak-Czerwenka[1], Andreas Seubert[3], Johann Heider[2,4#], Maciej Szaleniec[1*]

1 - Jerzy Haber Institute of Catalysis and Surface Chemistry Polish Academy of Sciences
2 - Faculty of Biology, Philipps-Universität Marburg, Marburg, Germany
3 - Faculty of Chemistry, Philipps-Universität Marburg, Marburg, Germany
4 - Center for Synthetic Microbiology, Philipps-Universität Marburg, Marburg, Germany
[*]maciej.szaleniec@ikifp.edu.pl
[#]heider@staff.uni-marburg.de

Abstract: Tungsten-dependent aldehyde oxidoreductases (AOR) catalyse the oxidation of aldehydes to acids and are the only known enzymes reducing non-activated acids using electron donors with low redox potentials. We report here that AOR from *Aromatoleum aromaticum* (AOR_{Aa}) catalyses the reduction of organic acids not only with low-potential Eu(II) or Ti(III) complexes but also with H_2 as an electron donor. Additionally, AOR_{Aa} catalyzes the H_2-dependent reduction of NAD^+ or benzyl viologen. The rate of H_2-dependent NAD^+ reduction equals to 10% of that of aldehyde oxidation, representing the highest H_2 turnover rate observed among the Mo/W enzymes. As AOR_{Aa} simultaneously catalyzes the reduction of acids and NAD^+ we designed a cascade reaction utilizing a NAD(P)H-dependent alcohol dehydrogenase to reduce organic acids to the corresponding alcohols with H_2 as the only reductant. The newly discovered W-hydrogenase side-activity of AOR_{Aa} may find applications in either NADH-recycling or conversion of carboxylic acids to more useful biochemicals.

Table of Contents

Table of Contents 2
Experimental Procedures 3
1. Chemicals 3
2. AOR_{Aa} plasmid construction 3
3. Recombinant AOR_{Aa} production and purification 3
4. Recombinant benzyl alcohol dehydrogenase (BaDH) production and purification 4
5. Coupled-enzyme assays 4
6. Steady-state activities of AOR_{Aa} in NAD^+/benzoate reduction 4
Controls for non-catalytic production of benzaldehyde 5
Control without hydrogen: 5
7. Analytical methods 5
b. HPLC method 2 5
d. LC-MS/MS identification of 4-hydroxybenzaldehyde 6
e. GC-MS identification of aldehydes 6
8. Calculations of hydrogen concentration from the solubility of gases in water 6
Results and Discussion 7
Biosynthesis and purification of recombinant AOR_{Aa} 7
pH optimum for NAD^+ reduction 8
Kinetic data for AOR_{Aa} hydrogenase activity 9
Reactivity of AOR_{Aa} with other acids 14
Cascade reactions 15
D_2/D_2O experiments 16
Potential applications 16
Detection of 4-hydroxybenzaldehyde 17
HPLC method 1 18
HPLC method 2 19
HPLC method 3 21
References 21
Author Contributions 21

Experimental Procedures

1. Chemicals

All organic acids (Sigma Aldrich) used in substrate screening and activity tests were solubilized by the addition of equimolar NaOH.

2. AOR_{Aa} plasmid construction

For the construct pAOR_2, structural genes *aorABC* (WP_011238651 - WP_011238653) were amplified via PCR from chromosomal DNA of *A. aromaticum* using the primers: forward 5'AAGCTCTTCAATGTGGAAATCGCTTCACATTGACCCCG' and reverse 5'AAGCTCTTCACCCTCGCACGAGCGCCATCGGTTGC3'. For the construct pAOR_1, the entire AOR_{Aa} operon (WP_011238651 - WP_011238653, WP_041646405 and WP_041646407) was amplified via PCR from chromosomal DNA using the primers: forward 5'AAGCTCTTCAATGTGGAAATCGCTTCACATTGACCC3' and reverse 5'AAGCTCTTCACCCCATTGCTTGCTGCGCTCGTCTG3'. The PCR was performed with Phusion™ High-Fidelity DNA-Polymerase and followed the established protocol (Thermo Fisher Scientific, Waltham, USA) with minor adaptations.

The amplified DNA fragments were ligated into pEntry via a simultaneous restriction/ligation reaction according to the instructions of the StarGate® cloning system (IBA Lifesciences, Göttingen, Germany). Sequences of the genes were validated by Sanger sequencing. The obtained plasmid was used in a second restriction/ligation reaction to move the genes into the expression vector pASG105_mob_ori(-) mobilizable vector based on the broad range vector pBBR[1,2]. The reaction was heat-inactivated (65°C, 20 min), 1 U T4 ligase (Thermo Fisher Scientific, Waltham, USA) and 0.5 mM ATP were added and the reaction was incubated further for 1 h at room temperature to enhance ligation. The obtained plasmids were transformed into a conjugation strain of *Escherichia coli* (WM3064[3]).

The final plasmids used were encoding the three subunits of AOR_{Aa} (AorABC: WP_011238651 - WP_011238653) behind an anhydrotetracycline (AHT)-inducible promotor with an N-terminal Twin-Strep-tag® fused to AorA. pAOR_1 (showed on Fig.S1) additionally contained the genes coding for the putative maturation factors AorD and AorE (WP_041646405 and WP_041646407). Both plasmids were then transferred from the donor *E. coli* strain to *A. evansii* by conjugation[2]. The recombinant *A. evansii* strain was grown anaerobically on a minimal medium (pH 7.8) with benzoate and nitrate as described in Sali *et al.*[2], with the addition of ampicillin and Na_2WO_4 to final concentrations of 100 µg/mL and 18 nM, respectively.

3. Recombinant AOR_{Aa} production and purification

Recombinant *A. evansii* cells were cultivated anaerobically either in 30 L fermenters or 2 L stoppered bottles with gentle shaking with periodic supplementation of nitrate and sodium benzoate (to concentrations of 10 mM and 4 mM, respectively), while monitoring nitrate and nitrite levels by test strips (Quantofix®, Machery-Nagel, Düren, Germany). The cultures were inoculated with 1% (v/v) of a preculture in the same medium, and cultivation was performed at 28 °C until the optical density (OD600) reached 0.6. At this point, the temperature was set to 18 °C and enzyme production was induced by the addition of AHT to a concentration of 200 ng /mL, while the culture was also supplemented with additional Na_2WO_4 to a final concentration of 10 µM. After 20 h incubation, the cells were harvested by centrifugation (4,500 x g, 1 h, 4 °C) and either frozen in liquid nitrogen and stored at -80°C, or processed further.

The cells were suspended in a stock buffer (100 mM Tris/HCl pH 8.0, 150 mM sodium chloride, 10% (w/v) glycerol) with 0.1 mg/mL DNase I and 0,1 mg/mL lysozyme and subsequently lysed by

ultrasonication (Sonics Vibra-Cell VCX500, intermittent cycle, 5 min, amplitude 40%, energy 150,000 J). Cell debris was separated by ultracentrifugation (40,000 x g, 1 h, 4 °C), and the cell-free extract was then applied to a Strep-tag® II affinity column (IBA Lifesciences, Göttingen, Germany). After rinsing with a stock buffer, the enzyme was eluted with elution buffer consisting of stock buffer with 10 mM desthiobiotin. The enzyme thus obtained was frozen at -80 °C and stored until use.

4. Recombinant benzyl alcohol dehydrogenase (BaDH) production and purification

The *bdh* gene of *A. aromaticum* strain EbN1 coding for a benzyl alcohol dehydrogenase (BaDH; ebA3166[4]) was amplified via PCR from chromosomal DNA using the primers 5'- AAGCTCTTCAATGAAGATTCAAGCCGCAGTAAC-3' (forward) and 5'- AAGCTCTTCACCCGGCGAGCCTAGGACCGGC-3' (reverse). The PCR product was cloned into the vector pASG-IBA103 (IBA Lifesciences, Göttingen, Germany). The resulting plasmid codes for a fusion protein of BaDH with a C-terminal Twin-Strep-tag®. BaDH was produced in *E. coli* BL21(DE3) which was grown in LB medium supplemented with 2 % (v/v) ethanol at 37 °C and induced at room temperature with added anhydrotetracycline as reported previously. Cells were harvested by centrifugation (40,000 x g, 30 min, 4 °C) and resuspended in three volumes of 100 mM Tris/HCl, 150 mM KCl pH 7.5, 10 % glycerol containing 0.1 mg/mL DNase I and 0,1 mg/mL lysozyme. The cell-free extract was prepared by sonification at 4 °C for 15 minutes, followed by ultracentrifugation (100,000 x g, 60 min). Cell-free extract with overproduced BaDH was applied on a Strep-tag® II affinity column, and further purification of the protein was performed as reported before[5].

Purified BaDH was checked for its activity by spectrophotometric assays and turned out to be specific for benzyl alcohol and only a few close chemical analogues, but accepted NAD^+ or $NADP^+$ almost equally well as electron acceptors. In addition, it also catalysed the reduction of benzaldehyde with either NADH or NADPH as an electron donor (manuscript in preparation).

5. Coupled-enzyme assays

The apparent kinetic parameters for the reduction of benzoic acid with H_2 were determined in a coupled assay with added BaDH, which was converting the produced benzaldehyde to benzyl alcohol in an NADPH-coupled reduction. The change in NADPH concentration was followed at 365 nm (ε = 3.5 mM^{-1} cm^{-1}) and benzaldehyde and benzyl alcohol production were quantitated by HPLC method 2. The assay was performed under 2.5% hydrogen in nitrogen (v/v) in the headspace at 30 ºC. The reaction mixture contained 100 mM citric buffer pH 5.5, 0.6 mM NADPH, BaDH (170 µg/mL) and AOR_{Aa} (0.03 mg/mL). The reaction was initiated by the addition of sodium benzoate to concentrations of 4-42 mM in the reaction mixture. The pH optimum of AOR_{Aa} for benzoate reduction with H_2 (2.5% v/v) was determined with the same coupled-enzyme assay, using 30 mM sodium benzoate and 100 mM citric buffer in the pH range of 4.5-6.0 and 50 mM K_2HPO_4/KH_2PO_4 buffer in the pH range of 6.0-7.0.

The cascade reactions with NAD^+ instead of NADPH were conducted under an anaerobic atmosphere at 30 °C and a pH of either 5.6 or 7.0 (100 mM citric acid/Na_2HPO_4 buffer) in a 2 mL cuvette containing 0.1 mM NAD^+, 30 mM sodium benzoate, 20 µg/mL AOR_{Aa} and 75 µg/mL BaDH. The concentration of NADH was followed spectrophotometrically at 340 nm while benzaldehyde and benzyl alcohol concentrations were followed by HPLC method 2.

6. Steady-state activities of AOR_{Aa} in NAD^+/benzoate reduction

AOR_{Aa} was tested for independent or competitive reduction of NAD^+ and benzoic acid with H_2 in 1.5 mL reactors at 30 °C under an anaerobic atmosphere with 0.02 mg/mL AOR. Following setups were tested: reduction with 2.5 % H_2 (v/v) of i) 0.1 mM NAD^+, ii) 1.0 mM NAD^+, iii) 30 mM sodium benzoate, iv) 30 mM sodium benzoate and 0.1 mM NAD^+, v) 30 mM sodium benzoate and 1 mM NAD^+ in 100 mM citric acid/Na_2HPO_4 buffer pH 7.0; and reduction with 2.5 % H_2 (v/v) of i) 0.1 mM NAD^+, ii) 1.0 mM NAD^+,

iii) 30 mM sodium benzoate, iv) 30 mM sodium benzoate and 1 mM NAD^+, v) 30 mM sodium benzoate and 0.1 mM NAD^+ in 100 mM citric acid/Na_2HPO_4 buffer pH 5.6. The change of NAD^+ concentration was measured by UV-vis at 340 nm while the concentration of benzaldehyde was followed by HPLC method 2. Each test was conducted in two repetitions. The reactions progress was followed for 30-80 min. The progress curves for NADH were fitted with linear function for the initial time of the reaction (0-30 s) while those for benzaldehyde were fitted with the burst-type equation $[P] = \Pi\,[1 - \exp(-k_b t)] + V_{ss}t$ provided in Origin Pro 2019b. The V_{ss} constant was used to calculate specific activities of the linear phase. The progress curve for benzaldehyde synthesis (Fig 2B) is shown for a reactor at pH 5.6 with 30 mM benzoate and 120 µM AOR_{Aa} under anaerobic conditions.

Controls for non-catalytic production of benzaldehyde

All mentioned controls were prepared in parallel with the enzyme assays reported using the same concentrations of reagents as in samples used for enzyme assays: 2.5 % H_2 (v/v) in the headspace, 30 mM benzoic acid in 100 mM citric acid/Na_2HPO_4 buffer pH 5.6 and:

a. no protein;
b. bovine serum albumin (BSA) instead of AOR_{Aa} - BSA (Sigma-Aldrich, Saint Louis, USA) to a concentration of 0.02 mg/mL in the reaction mixture
c. AOR_{Aa} (2mg/ml, 200 ul) after inactivation by incubation at 95 °C and centrifugation- the supernatant was tested for the presence of aldehyde, precipitated protein was mixed with reagents to a final concentration of protein 0.02 mg/ml.

Samples were taken from reactions after 1 h and were examined according to HPLC method 2. None of the above-mentioned controls showed the presence of any benzaldehyde or benzyl alcohol.

Control without hydrogen:

A capped vial with 2 mL of 30 mM benzoic acid, 100 mM citric buffer, pH 5.6, was flushed with nitrogen to get rid of any hydrogen (mixture prepared in the glovebox). AOR_{Aa} was added to a concentration of 0.02 mg/mL in the reaction mixture and samples were collected after 1 h. Samples taken from reactions after 1 h were examined according to HPLC method 2. None of these controls showed the presence of any benzaldehyde or benzyl alcohol.

7. Analytical methods

a. HPLC method 1

Two samples were collected for each time point and the reactions were stopped by mixing the samples with acetonitrile in a 1:1 (v:v) ratio. The samples (30 µL per injection) were analyzed) on an Agilent 1100 HPLC, using a LUNA® 3u C8 column (100Å, 150x4.6 mm, 3µm bead size, Phenomenex, USA) at 40 ºC at 0.75 mL/min flow rate in isocratic mode with a water/ACN 65/45 (v/v) mobile phase. The runs were monitored at 245 nm using a DAD detector, and peaks were quantitated by integration and comparison to the calibration curve (see Fig. S11) with the respective standards.

b. HPLC method 2

The concentrations of benzaldehyde and/or benzyl alcohol were determined using HPLC-DAD methods. Two 100 µL samples were collected for each time point and the reaction was stopped by mixing the samples with acetonitrile in a 7:5 (v/v) ratio. Aliquots of 2 µL of the samples were analysed with Agilent 1260 UHPLC equipped with DAD using a ZORBAX 300 SB-C18 column (RRHD, 2.1 x 50mm, 1.8 µm, Agilent, USA) at 30 °C and 0.2 mL/min flow rate in the isocratic mode 70/30 H_2O/ACN with a 0.1% formic acid mobile phase. Each sample was analyzed in triplicate. Benzyl alcohol was monitored

at 210 nm (RT:1.7 min), and benzaldehyde at 250 nm (RT: 3.4 min). The quantitation of both compounds was conducted based on external standard calibration curves (Fig. S13) and both LOD and LOQ were about 5 and 15 µM (Table S4), respectively.

c. HPLC method 3

The method was used for the detection of (*R*)-1-phenylethanol.

The product concentrations were determined with HPLC using an Agilent 1100 system equipped with a DAD detector. The separations were performed on the Ascentic® RP-Amide Express column (75 mm × 4.6 mm, 2.7 µm) at 40 ℃ at the flow rate of 1 mL/min and injection volumes of 20 µL mobile phase H_2O (A) and CH_3CN(B) as a mobile phase. The following gradient program was used: 0-0.5 min 20% (v/v) B, 0.5 to 0.8 min gradient to 35% B, 0.8 to 4.2 min isocratic 35%. The quantitation of substrate was conducted at 210 nm against external standard calibration (Fig. S13).

d. LC-MS/MS identification of 4-hydroxybenzaldehyde

The reactions were stopped by the addition of acetonitrile 1:1 to the reaction mixture and centrifugation. The samples (5µL) were analysed by an Agilent 1260 UHPLC equipped with DAD using a ZORBAX 300 SB-C18 column at 30 °C and 0.2 mL/min flow rate in the isocratic mode 85/15 H_2O/MetOH with a 0.1% formic acid mobile phase. The product (4-hydroxybenzaldehyde) was eluted at 2.4 min (see Fig. S11) and was detected by LC-MS/MS identification by a qualifier product ion (123.0 → 77.1) and quantifier (123.0 → 95) (details of MS method in Table S4).

e. GC-MS identification of aldehydes

The reactions were stopped by the addition of 1 % of 1M HCl to precipitate the enzyme, centrifuged and neutralized. Then, the aldehydes were separated from the reaction mixture by solid-phase extraction (SPE): i) in the case of the reaction mixture with benzoate, Strata-X 33µm Polymeric Reversed Phase 100 mg/3 mL columns (Phenomenex) were used, ii) in case of the other substrates, Reversed-Phase Speedisk™ Octadecyl (C18) columns (Avantor™ BAKERBOND). The columns were equilibrated before sample application with 1 mL methanol and 1 mL water, and all liquids were applied at a 2 mL/min flow rate. The analytes were extracted from the air-dried SPE columns with 500 µL of methanol and eluents were stored anaerobically at -20 °C until further examination.

The analyses were performed using a Trace GC/DSQII-MS single quad GC-EI(+)-MS using a TR-5MS column (30 m x 0.25mm ID x 0.25 µm film) with helium as a carrier gas at the flow rate of 1.2 mL/min. Samples of 1 µL were injected in a constant temperature (CT) splitless mode at 250 °C and separated according to the following temperature program: 60°C hold for 2 min, gradient from 60 to 200 °C at 10 °C/min, followed by a gradient from 200 to 290 °C at 30 °C/min and 5 min hold at 290 °C. The MS detector operated in positive ion scanning mode with a mass range of 35-200 m/z, at the electron energy of 70 eV. The ion source and transfer line temperatures were set at 230 °C and 250 °C, respectively. Compounds were identified based on the NIST library or against MS spectra of an external standards library (analysis of standards in the above method was carried out before analysis of samples).

8. Calculations of hydrogen concentration from the solubility of gases in water

For calculating apparent kinetic parameters of hydrogen, partial pressures were converted into concentration by assuming a solubility coefficient of q= 1.47 x 10^{-4} g hydrogen in 100 g of water at 30 °C and 1 atmosphere [6].

Results and Discussion

Biosynthesis and purification of recombinant AOR$_{Aa}$

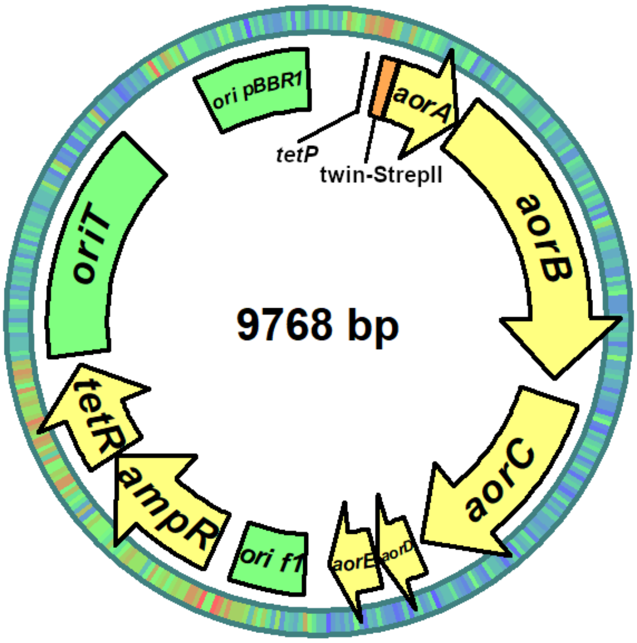

Figure S1. Plasmid map of pAOR_1. This plasmid was used for the recombinant production of AOR. *ori* pBBR1, broad host range origin of replication; *tetP* promoter, AHT inducible promotor; *aorABC* genes for the subunits of AOR; *aorD* and *aorE*, potential genes for maturation factors; *ori* f1, f1 phage origin of replication; *ampR*, beta-lactamase gene; *tetR*, tetracycline repressor gene.

pH optimum for NAD$^+$ reduction

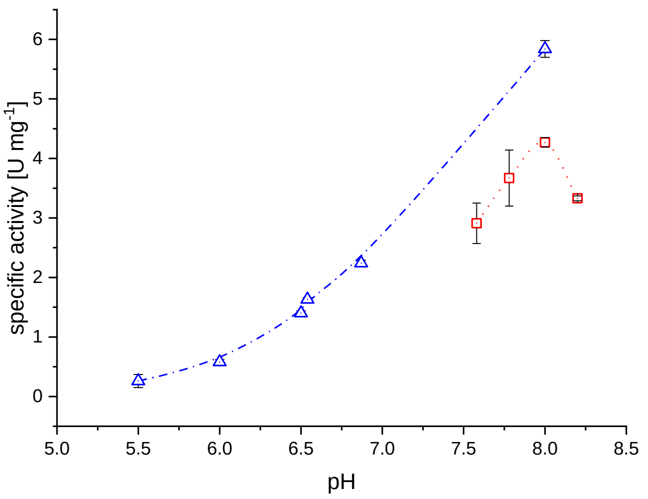

Figure S2. pH-dependence of AOR$_{Aa}$ activity for NAD$^+$ reduction with H_2 as an electron donor. Blue triangles – phosphate buffer, red squares – Tris/HCl buffer; error bars represent standard deviations.

Kinetic data for AOR_{Aa} hydrogenase activity

A

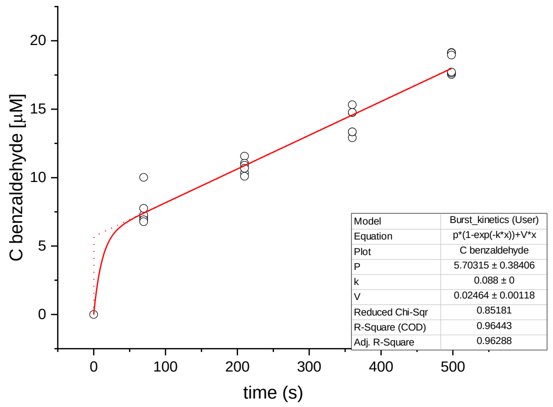

B

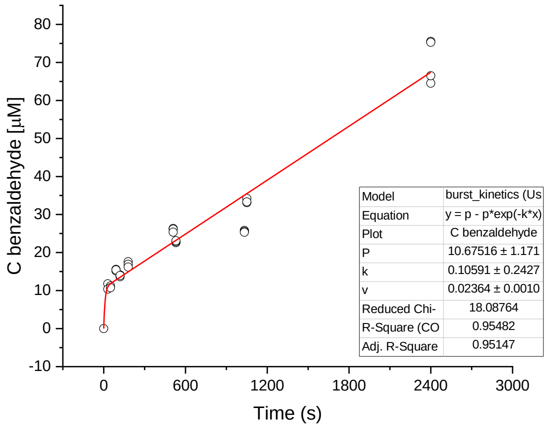

C

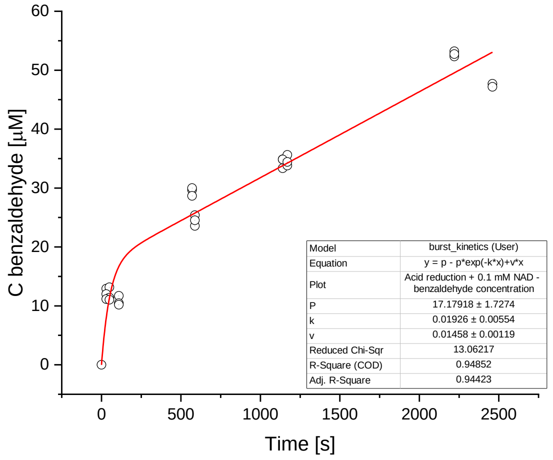

D

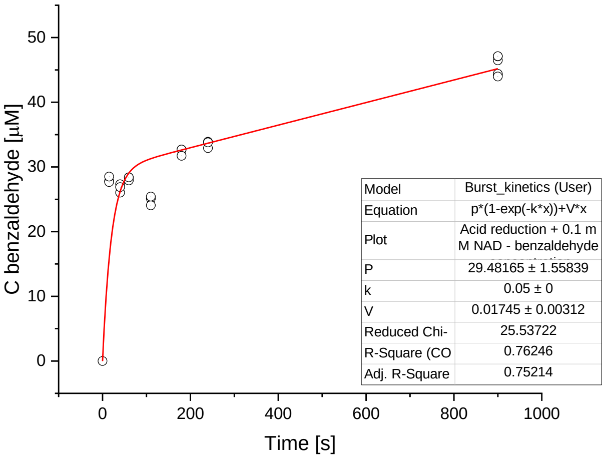

E

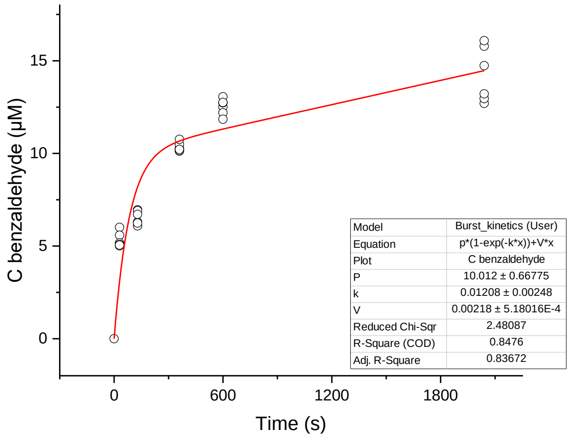

F

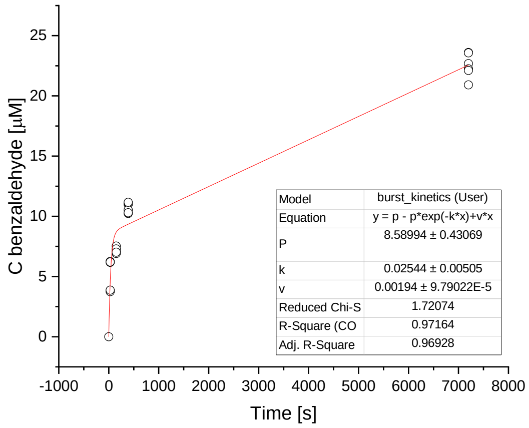

G H

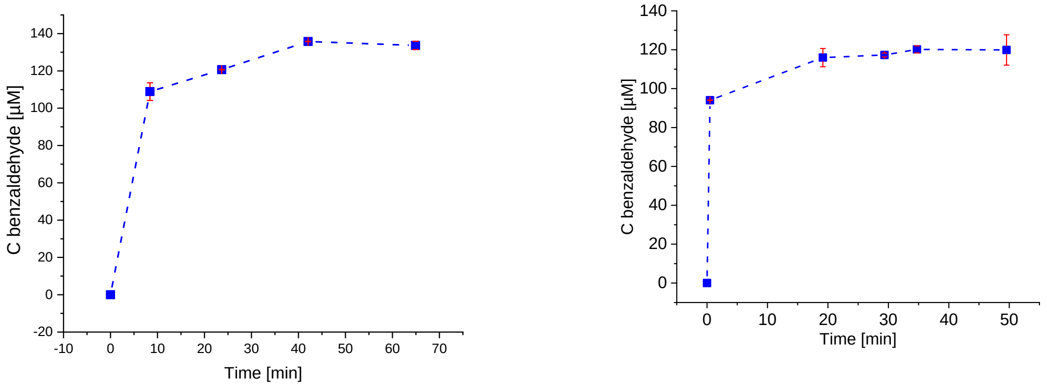

Figure S3. Progress curves of benzaldehyde synthesis from benzoate and H_2 (2.5% in headspace) with 60 nM AOR, in 100 mM citric acid/Na_2HPO_4 buffer at pH 5.6 under the following conditions: A and B) with 30 mM benzoate, C and D) with 30 mM benzoate and 0.1 mM NAD^+, E) and F) with 30 mM benzoate and 1.0 mM NAD^+; G) and H) as in A), with double concentration of AOR_{Aa} (120 nM). The measured values (circles) of A-F were fitted with the burst-type equation $[P] = \Pi\ [1 - \exp(- k_{b}t)] + V_{ss}\, t$ (red lines). Benzaldehyde concentrations were quantified by HPLC method 2.

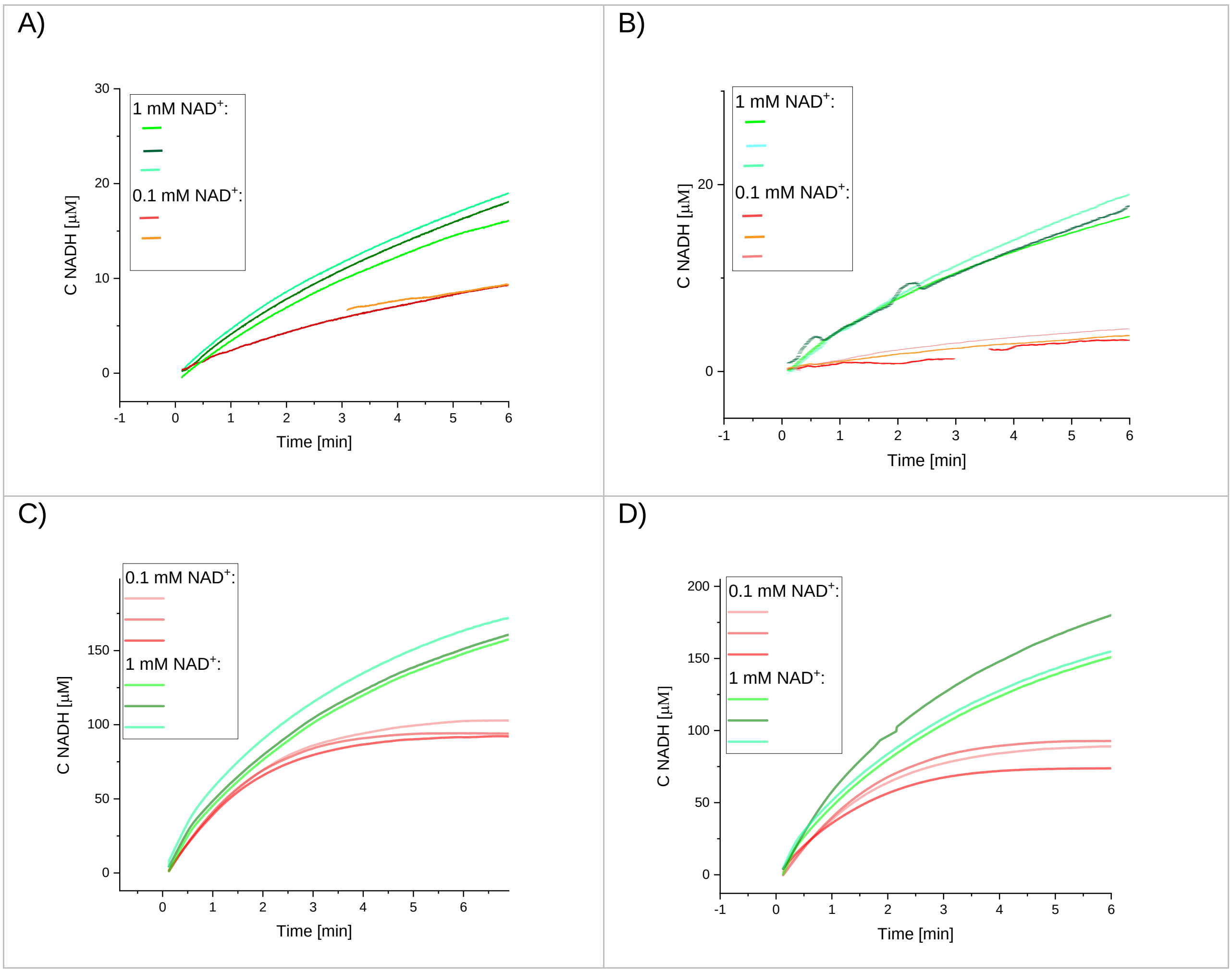

Figure S4. Progress curves of NADH synthesis from NAD^+ and H_2 (2.5% in headspace) with 60 mM AOR, in in 100 mM citric acid/Na_2HPO_4 buffer. The assays with 0.1 mM NAD^+ (red lines) or 1 mM NAD^+ (green lines) at following conditions: A) pH 5.6, B) pH 5.6 with 30 mM benzoate, C) pH 7.0, D) pH 7.0 with 30 mM benzoate.

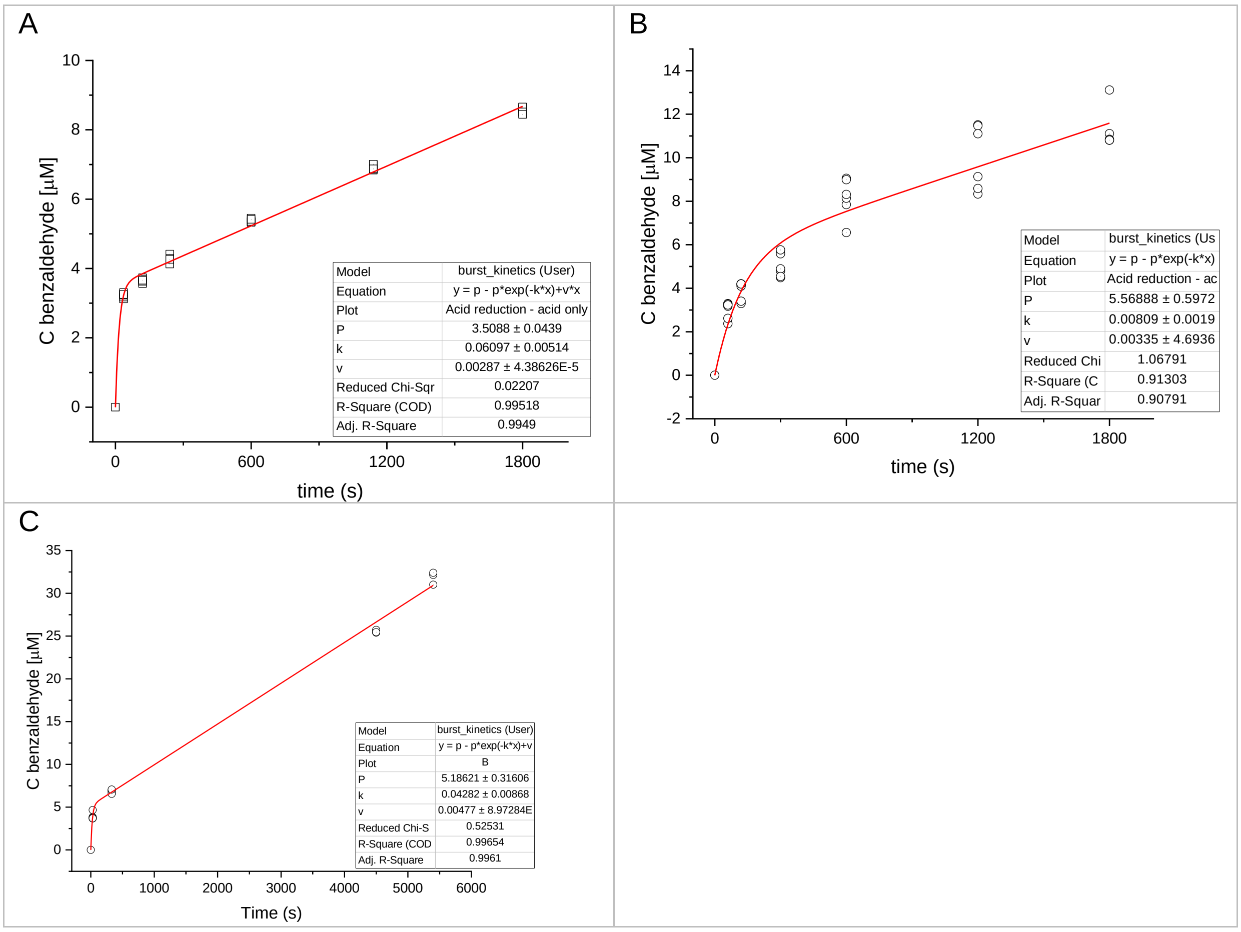

Figure S5. Progress curves of benzaldehyde synthesis from benzoate and H_2 (2.5% in headspace) with 60 nM AOR, in 100 mM citric acid/Na_2HPO_4 buffer at pH 7.0 under the following conditions: A and B) with 30 mM benzoate, C) with 30 mM benzoate and 0.1 mM NAD^+ The measured values (circles) of A-F were fitted with the burst-type equation $[P] = \Pi\ [1- \exp(- k_{bt})] + V_{ss}t$ (red lines). Benzaldehyde concentrations were quantified by HPLC method 2.

Table S1. Specific activities of benzoate reduction by AOR_{Aa} in individual experiments. Calculations of specific activities were based on the V_{ss} values obtained from the burst kinetics models.

Reaction set-up	Specific activity of AOR_{Aa} [mU/mg]	
	pH 5.6	pH 7.0
Benzoic acid	73.92	8.61
	70.92	10.05
0.1 mM NAD^+ + benzoate	52.35	14
	43.74	-
1 mM NAD^+ + benzoate	6.54	n.d.
	5.82	n.d.

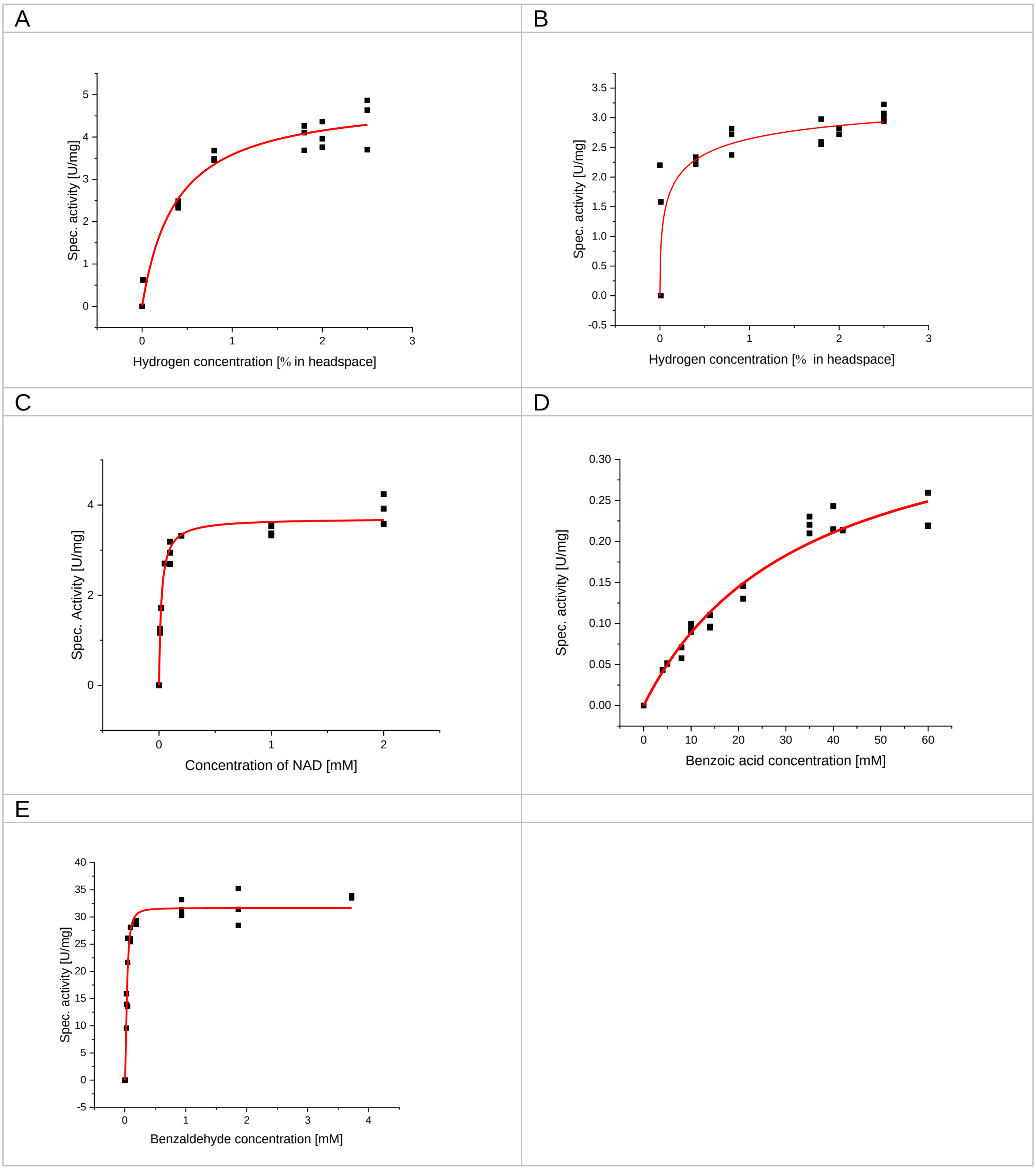

Figure S6. Michaelis-Menten kinetic curve fits for AOR$_{Aa}$ activities: A) variable H_2 concentrations at constant BV^{2+} (1 mM); B) variable H_2 concentrations at constant NAD^+(1 mM); C) variable NAD^+ concentration at constant H_2 (2.5 %), D) variable benzoate concentrations at constant H_2 (2.5 %); E) variable benzaldehyde concentrations at constant NAD^+ (1 mM).

Reactivity of AOR_{Aa} with other acids

Table S2. Identification of products based on LC-MS/MS or GC-MS detection of appropriate peaks. For more details on the 4-hydroxybenzaldehyde detection see Table S4, Fig. S10 and S11.

Substrate	Product	LC-MS/MS Precursor ion and derived product ions [m/z]	GC-MS signals of detected product [m/z]
benzoic acid	benzaldehyde		106 $[M]^+$<br>105 $[M-H]^+$<br>77 $[M-CHO]^+$<br>51
4-hydroxybenzoic acid	4-hydroxybenzaldehyde	$[M+H]^+$ 123<br>123.0 → 77.1<br>123.0 →95	
trans-cinnamic acid	*trans*-cinnamaldehyde		132 $[M]^+$<br>131 $[M-H]^+$<br>103 $[M-CHO]^+$<br>77 $[M-C_2H_2CHO]^+$<br>51
phenylacetic acid	phenylacetaldehyde		120 $[M]^+$<br>91 $[M-CHO]^+$<br>77 $[M-CH_2CHO]^+$<br>65
octanoic acid	octanal		100 $[M+H-CHO]^+$<br>84 $[M-C_3H_8]^+$<br>110, 69, 56, 44
nicotinic acid	3-pyridine-carboxaldehyde		107 $[M]^+$<br>106 $[M-H]^+$<br>78 $[M-CHO]^+$<br>51
vanillic acid	vanillin		152 $[M]^+$<br>151 $[M-H]^+$<br>123 $[M-CHO]^+$<br>109, 81

Cascade reactions

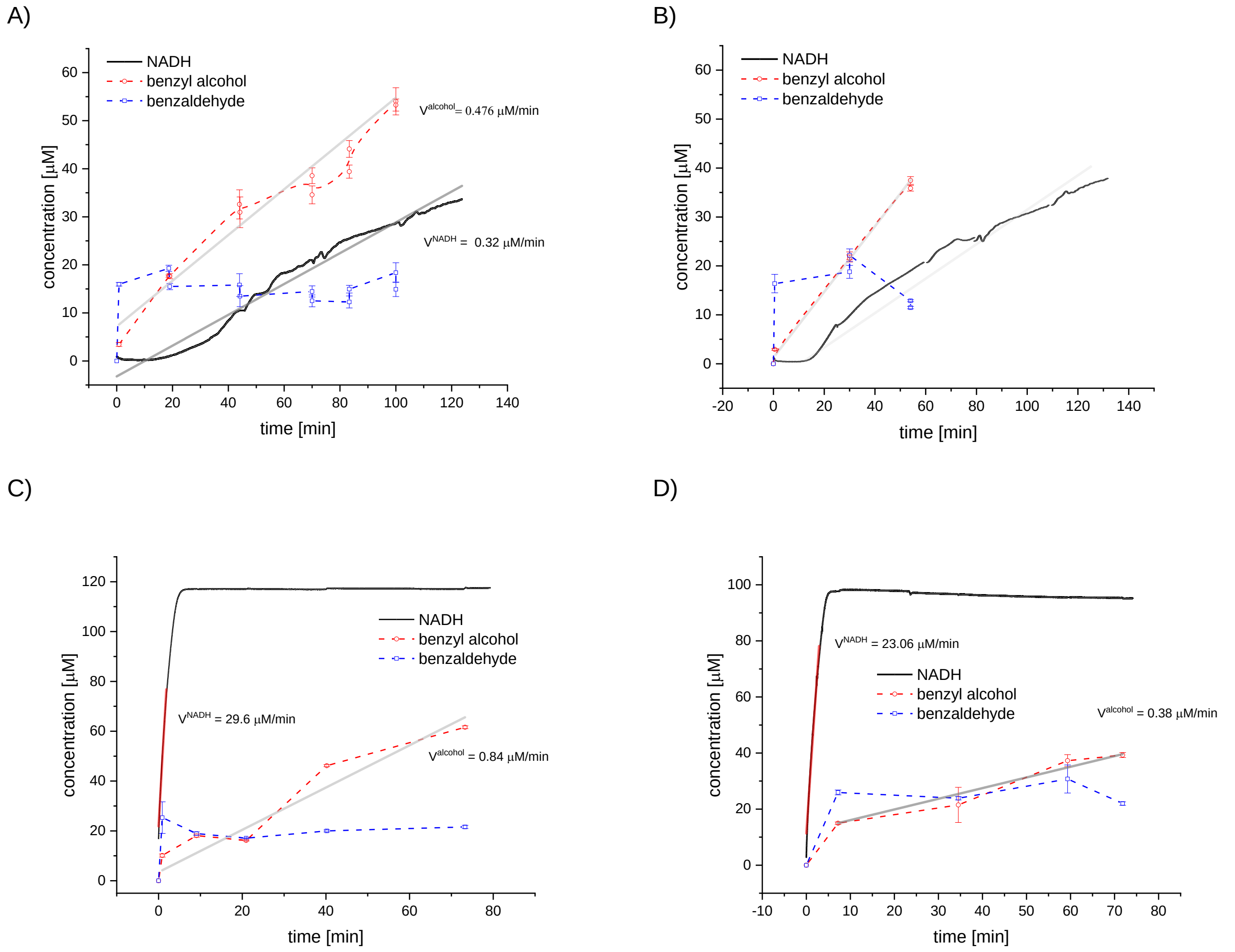

Figure S7. Progress curves of coupled reactions of benzoate reduction to benzaldehyde and benzyl alcohol**.** All assays contained 0.1 mM NAD^+, 30 mM benzoate, 2.5% H_2, 60 nM AOR, 500 nM BaDH and were incubated at 30 ºC. A and B) two repetitions at pH 5.6, C and D) two repetitions at pH 7.0. Product accumulation is shown in black (NADH), red (benzyl alcohol), and blue (benzaldehyde). Rates for NADH and benzyl alcohol accumulation were fitted linearly and indicated by grey lines; the error bars represent SD.

D_2/D_2O experiments

Table S3. Results of GC-MS analysis of benzaldehyde product for reactions conducted with isotope labelled hydrogen (D_2) or in 95% D_2O.

Mass signal [m/z]	Standard		H_2/H_2O	H_2/D_2O	D_2/H_2O	H_2/Eu(II)/D_2O
	benzaldehyde	d^1-benzaldehyde				
			**absolute abundance**			
105	49823744	5815266	37013	38490	98622	129194
106	48108800	567066	28922	25033	77815	31715
107	3835817	5840551	2230	5297	6542	83429
108	222776	446076	90	918	1039	7705
			**relative abundance**			
105	100.00%	99.6%	100.00%	100.0%	100.0%	100.0%
106	96.56%	9.7%	78.14%	65.0%	78.9%	24.5%
107	7.70%	100.0%	6.02%	13.8%	6.6%	64.6%
108	0.45%	7.6%	0.24%	2.4%	1.1%	6.0%
d^1-benzaldehyde [%]	**0.00%**	**100.0%**	**0.00%**	**27.0%**	**8.4%**	**76.7%**

Potential applications

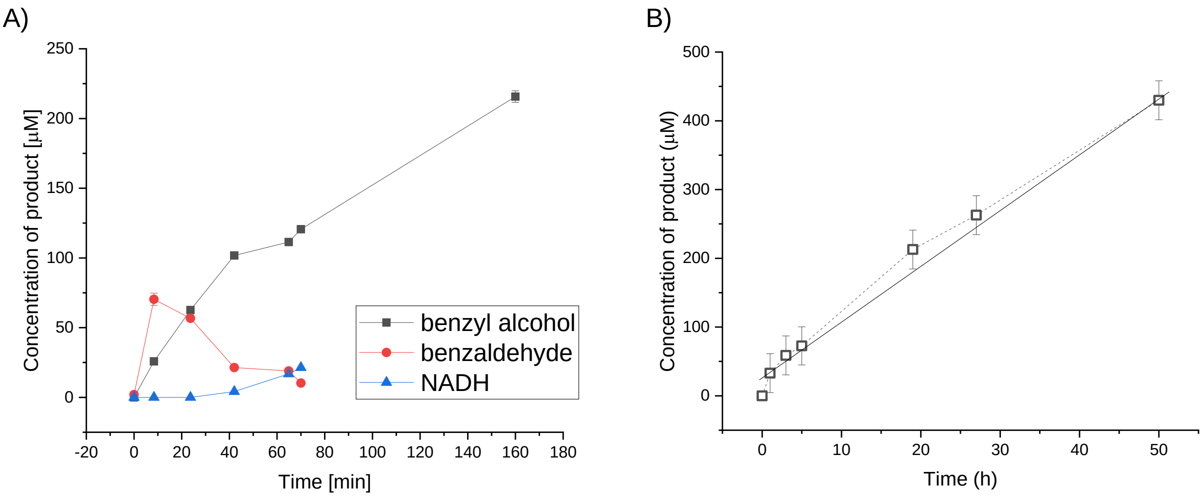

Figure S8. Progress curves of coupled reactions A) production of benzyl alcohol from 30 mM benzoate, 0.1 mM NAD^+ and 2.5 % H_2 at pH 5.5 catalyzed by 120 nM AOR*Aa* and 500 nM BaDH; B) synthesis of (*R*)-1-phenylethanol from acetophenone by R-HPED where AOR*Aa* was used to recycle NADH with 2.5 % H_2; analytical repetition average, the error bars represent SD.

Detection of 4-hydroxybenzaldehyde

Table S4. HPLC and MS/MS parameters of the method used for detection of 4-hydroxybenzaldehyde in the reaction mixture.

MS Parameters for 4-hydroxybenzaldehyde method	
Ionization mode	ESI
Gas temperature [ºC]	300
Gas flow [L/min]	11
Nebulizer pressure [ºC]	25
Sheath gas temperature [ºC]	300
Sheath gas flow [L/min]	10
Capillary voltage [V]	3000
Nozzle voltage [V]	1000

Detected chemical	Precursor ion [m/z]	Product ion [m/z]	Fragmentor	CE	CAV	Polarity
4-hydroxy benzaldehyde	123	95	100	9	7	Positive
	123	77	100	21	7	Positive

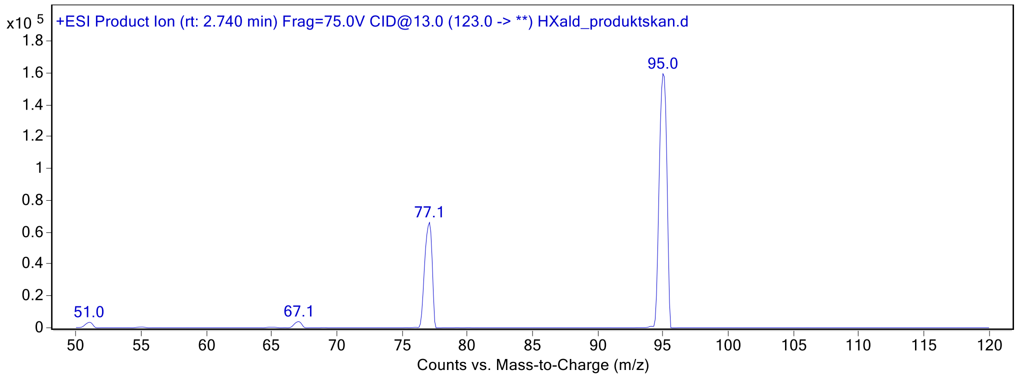

Figure S9. Product ion scan (positive) for ion 123 m/z, the parent ion in fragmentation of 4-hydroxybenzaldehyde.

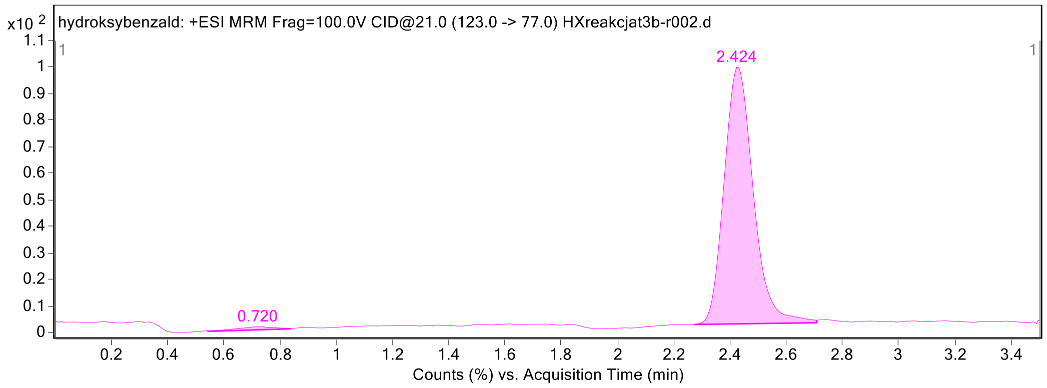

Figure S10. Chromatogram of the reaction mixture with shown quantifier transition (123 m/z -> 95.1 m/z) signal (percent of counts on the detector), peak at the retention time of 2.4 min corresponds to 4-hydroxybenzaldehyde.

HPLC method 1

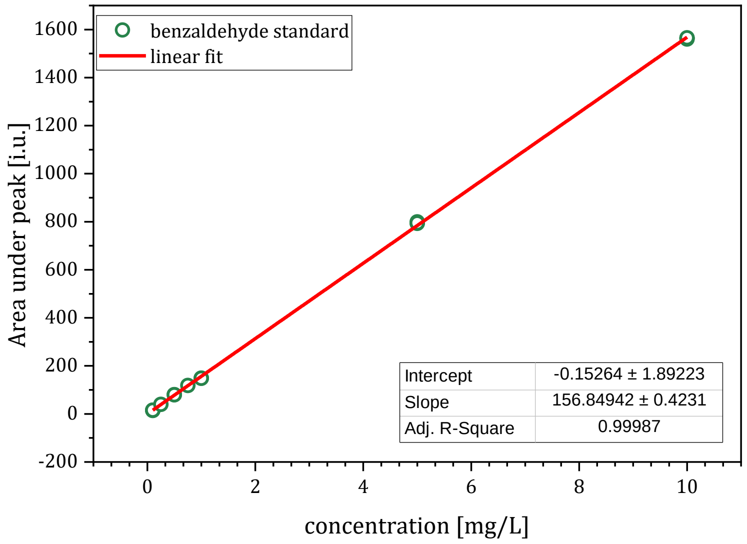

Figure S11. Calibration curve for detection of benzaldehyde on 245 nm signal, in a method on Agilent 1100 HPLC using the LUNA® 3μM C8 column (100 Å, 150x4.6 mm). All points were collected in triplicate.

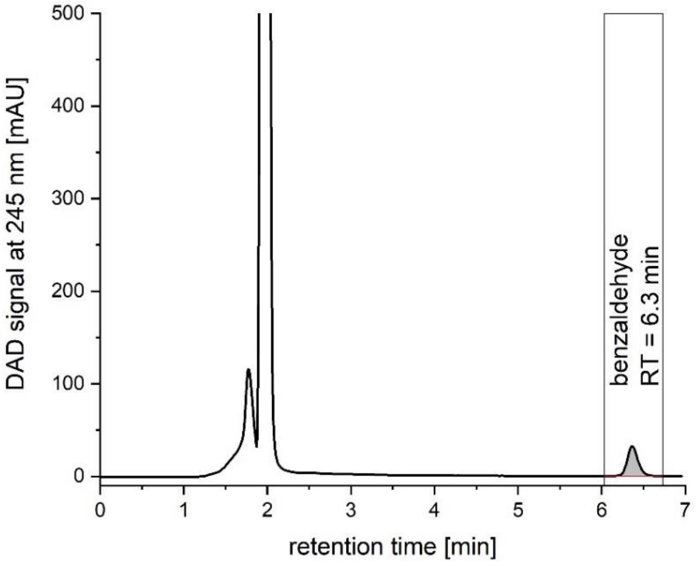

Figure S12. Chromatogram of the reaction mixture collected with DAD at 245 nm according to method 1, peak with the retention time (RT) of 2.1 min corresponds to benzoic acid, 6.3 min to benzaldehyde.

HPLC method 2

A)

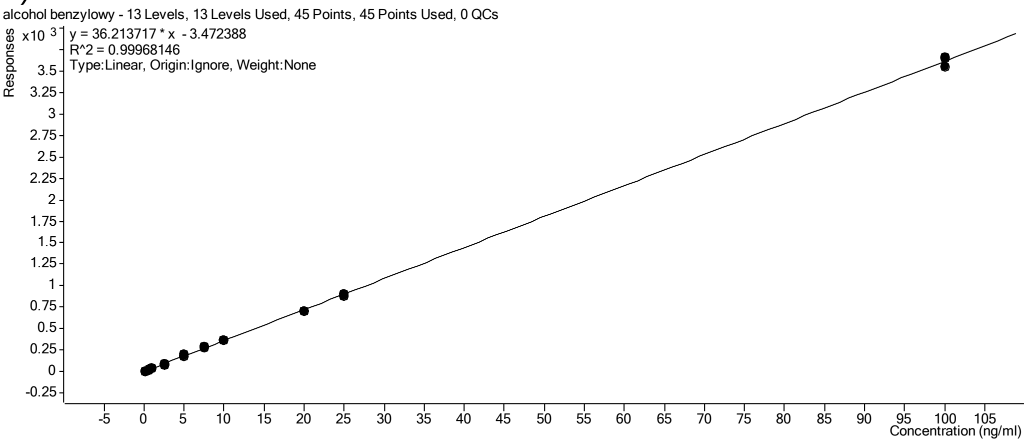

B)

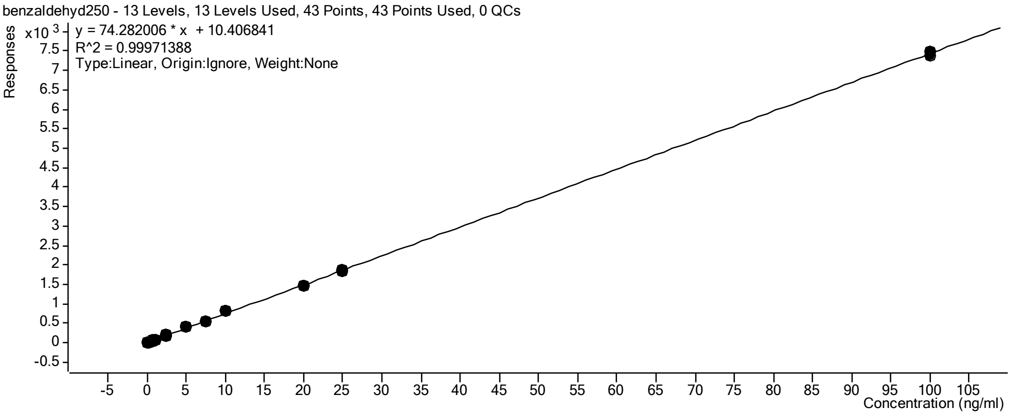

Figure S13. A) DAD (210 nm) calibration curve for benzyl alcohol quantitation, b) DAD (250 nm) calibration curve for benzaldehyde quantitation on UHPLC method. Values for concentration are in ng/ml. Each concentration point was measured in triplicate.

Table S5. Limit of detection (LOD) and limit of quantitation (LOQ) for the UHPLC method for benzaldehyde and benzyl alcohol detection. Calculated from the standard deviation of intercept.

Type of limit	Concentration of benzyl alcohol in reaction [µM]	Concentration of benzaldehyde in reaction [µM]
**LOD**	4.84	4.31
**LOQ**	14.65	13.06

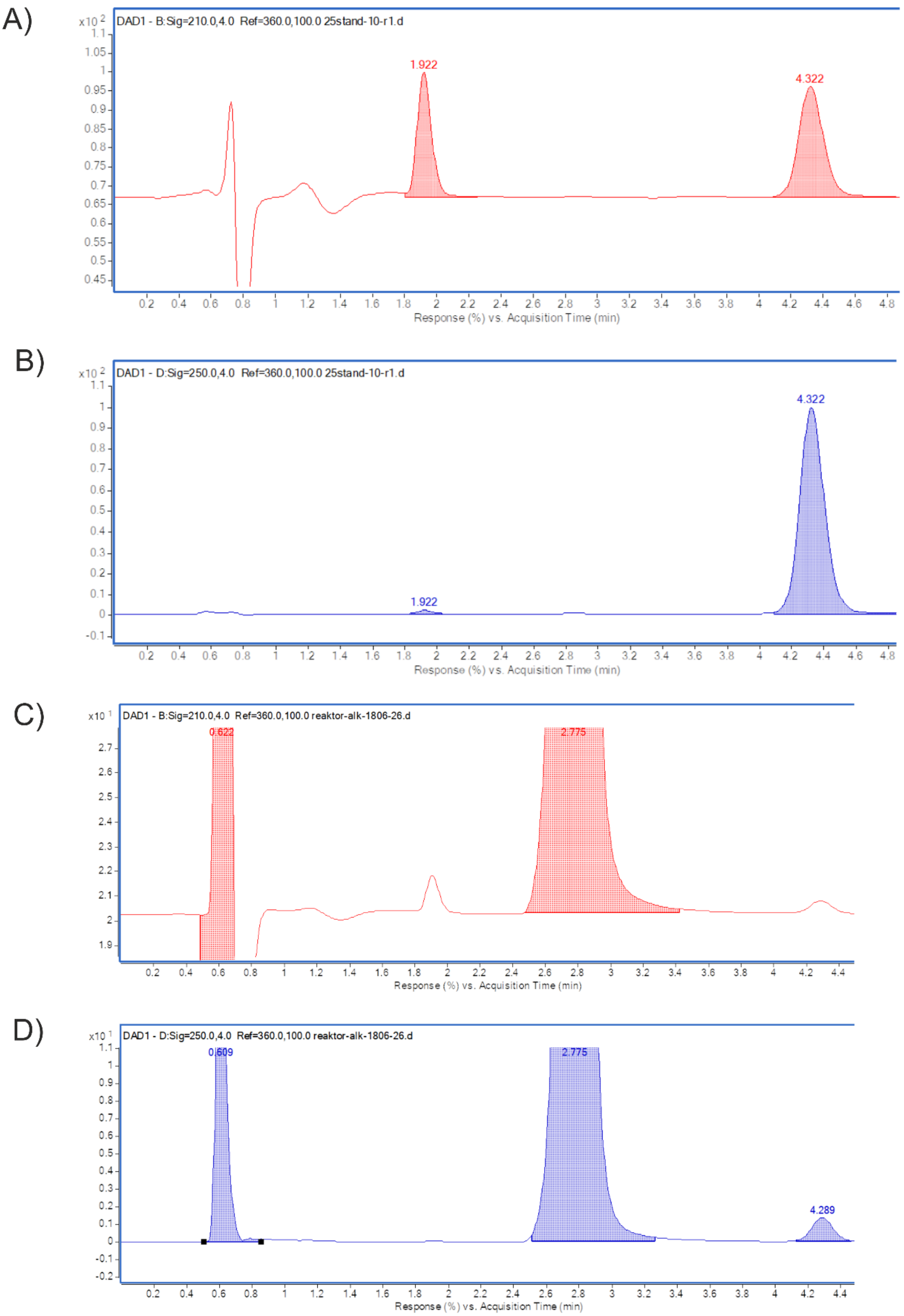

Figure S14. Chromatogram of A) benzyl alcohol (RT:1.9 min) and benzaldehyde (RT: 4.3 min) standards at 210 nm, peak at retention time 0.6 min corresponds to dead time of the system; B) both standards at 250 nm; an example of reaction mixture C) at 210 nm, peak at RT 1.7 min - benzyl alcohol, RT 2.8 min - benzoic acid; D) reaction mixture at 250 nm.

HPLC method 3

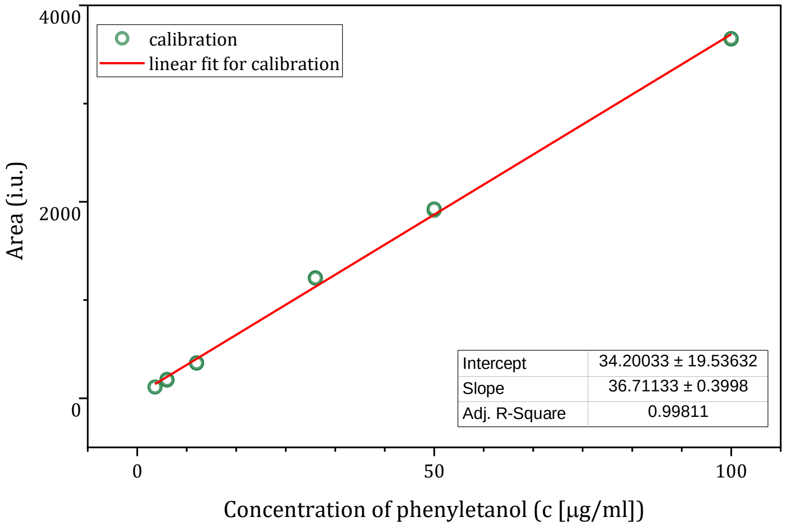

Figure S15. Calibration curve for (R)-1-phenylethanol quantitation in HPLC method 3 for DAD signal (210 nm). All points were collected in triplicate.

References

(1) Kovach, M. E.; Phillips, R. W.; Elzer, P. H.; Roop, R. M.; Peterson, K. M. PBBR1MCS: A Broad-Host-Range Cloning Vector. *Biotechniques* **1994**, *16* (5), 800–802.

(2) Salii, I.; Szaleniec, M.; Zein, A. A.; Seyhan, D.; Sekuła, A.; Schühle, K.; Kaplieva-Dudek, I.; Linne, U.; Meckenstock, R. U.; Heider, J. Determinants for Substrate Recognition in the Glycyl Radical Enzyme Benzylsuccinate Synthase Revealed by Targeted Mutagenesis. *ACS Catalysis* **2021**, *11* (6), 3361–3370. https://doi.org/10.1021/acscatal.0c04954.

(3) Dehio, C.; Meyer, M. Maintenance of Broad-Host-Range Incompatibility Group P and Group Q Plasmids and Transposition of Tn5 in Bartonella Henselae Following Conjugal Plasmid Transfer from Escherichia Coli. *Journal of Bacteriology* **1997**, *179* (2), 538–540. https://doi.org/10.1128/jb.179.2.538-540.1997.

(4) Rabus, R.; Kube, M.; Heider, J.; Beck, A.; Heitmann, K.; Widdel, F.; Reinhardt, R. The Genome Sequence of an Anaerobic Aromatic-Degrading Denitrifying Bacterium, Strain EbN1. *Archives of Microbiology* **2005**, *183* (1), 27–36. https://doi.org/10.1007/s00203-004-0742-9.

(5) Schühle, K.; Nies, J.; Heider, J. An Indoleacetate-CoA Ligase and a Phenylsuccinyl-CoA Transferase Involved in Anaerobic Metabolism of Auxin. *Environmental Microbiology* **2016**, *18* (9), 3120–3132. https://doi.org/10.1111/1462-2920.13347.

(6) *Lange's Handbook of Chemistry*, 12th ed.; Dean, J. A., Ed.; McGraw-Hill Inc.: New York, 1979; Vol. 68. https://doi.org/10.1002/jps.2600680645.

Author Contributions

A.W. did biochemical assays, kinetics experiments, cascades, deuteration, screened substrate screening, biotech application, developed HPLC and LC-MS/MS methods and conducted analytics, wrote and edited manuscript and SI, provided funding; D. H. developed AOR expression system, did biochemical assays and activity staining with AOR, conducted experiments with H_2 evolution; Y.G. developed recombinant BaDH production and did biochemical assays; J. K-C. did GC-MS and wrote method description, A. S. did ICP-MS elemental analysis, J. H. supervised the work of A.W., D.H., Y.G, designed experiments, analysed data, wrote and edited manuscript and provided funding; M. S. – supervised the work of A. W. and J. K-C, designed experiments, analysed data, wrote and edited manuscripts, prepared the figures. All authors revised the manuscript.
